# Inhibition of Shikimate Kinase from Methicillin-Resistant *Staphylococcus aureus* by Benzimidazole Derivatives. Kinetic, Computational, Toxicological, and Biological Activity Studies

**DOI:** 10.3390/ijms25105077

**Published:** 2024-05-07

**Authors:** Lluvia Rios-Soto, Alicia Hernández-Campos, David Tovar-Escobar, Rafael Castillo, Erick Sierra-Campos, Mónica Valdez-Solana, Alfredo Téllez-Valencia, Claudia Avitia-Domínguez

**Affiliations:** 1 Facultad de Medicina y Nutrición, Universidad Juárez del Estado de Durango, Av. Universidad y Fanny, Anitúa S/N, Durango 34000, Mexico; lluviarios.soto@gmail.com; 2 Departamento de Farmacia, Facultad de Química, Universidad Nacional Autónoma de México, México City 04510, Mexico; hercam@unam.mx (A.H.-C.); dvdtxo@gmail.com (D.T.-E.); rafaelc@unam.mx (R.C.); 3 Facultad de Ciencias Químicas, Universidad Juárez del Estado de Durango, Av. Artículo 123 S/N Fracc. Filadelfia, Gómez Palacio 35010, Mexico; ericksier@ujed.mx (E.S.-C.); valdezandyval@gmail.com (M.V.-S.)

**Keywords:** methicillin-resistant *Staphylococcus aureus*, antimicrobial resistance, shikimate pathway, shikimate kinase, enzyme inhibition, molecular dynamics

## Abstract

Antimicrobial resistance (AMR) is one of the biggest threats in modern times. It was estimated that in 2019, 1.27 million deaths occurred around the globe due to AMR. Methicillin-resistant *Staphylococcus aureus *(MRSA) strains, a pathogen considered of high priority by the World Health Organization, have proven to be resistant to most of the actual antimicrobial treatments. Therefore, new treatments are required to be able to manage this increasing threat. Under this perspective, an important metabolic pathway for MRSA survival, and absent in mammals, is the shikimate pathway, which is involved in the biosynthesis of chorismate, an intermediate for the synthesis of aromatic amino acids, folates, and ubiquinone. Therefore, the enzymes of this route have been considered good targets to design novel antibiotics. The fifth step of the route is performed by shikimate kinase (SK). In this study, an in-house chemical library of 170 benzimidazole derivatives was screened against MRSA shikimate kinase (SaSK). This effort led to the identification of the first SaSK inhibitors, and the two inhibitors with the greatest inhibition activity (**C1** and **C2**) were characterized. Kinetic studies showed that both compounds were competitive inhibitors with respect to ATP and non-competitive for shikimate. Structural analysis through molecular docking and molecular dynamics simulations indicated that both inhibitors interacted with ARG113, an important residue involved in ATP binding, and formed stable complexes during the simulation period. Biological activity evaluation showed that both compounds were able to inhibit the growth of a MRSA strain. Mitochondrial assays showed that both compounds modify the activity of electron transport chain complexes. Finally, ADMETox predictions suggested that, in general, **C1** and **C2** can be considered as potential drug candidates. Therefore, the benzimidazole derivatives reported here are the first SaSK inhibitors, representing a promising scaffold and a guide to design new drugs against MRSA.

## 1. Introduction

Nowadays, we are facing one of the biggest threats in modern times, antimicrobial resistance (AMR) [1,2,3]. This problem has arisen since the creation of antibiotics, and it has become one of the leading causes for complicated infections, which result in prolonged hospital stays and often lead to increased mortality among patients [4]. It was estimated that in 2019, 1.27 million deaths occurred around the globe due to AMR. Over time, efforts have been made in order to diminish and control AMR [5]. However, due to population misinformation and the occurrence of coinfection in patients with COVID-19 during the pandemic years, substantial increase in antibiotic use appeared [6,7,8,9].

Now AMR is considered a hidden pandemic that is looming behind us and threatening our future [10]. Additionally, surgical and invasive procedures are still the norm in hospital settings, which continuously increase expanded antibiotic use, worsening the risk of higher antimicrobial resistance emergence [11]. As a result, patients and, surprisingly, the general population are becoming infected over short periods of time in a constant manner, resulting in increased use of antimicrobial drugs.

*Staphylococcus aureus* is a pathogen considered a high priority by the World Health Organization [12]. Furthermore, it is highly implicated in skin and soft tissue infections and recently in coinfections in COVID-19 patients [13,14], where coinfection with COVID-19 and *Staphylococcus aureus* demonstrates a higher patient mortality during hospital admission [15]. Methicillin-resistant *Staphylococcus aureus *(MRSA) strains have proven to be resistant to most of the actual antimicrobial treatments, which has complicated the establishment of treatments for patients carrying infections by this bacterium. Therefore, new treatments are required to be able to manage this increasing threat.

Under this perspective, an important metabolic pathway for MRSA is the shikimate pathway. This route comprises seven enzymatic reactions that utilize erythrose-4-phosphate, which is derived from the pentose phosphate pathway, and phosphoenolpyruvate, which is generated by glycolysis, to obtain chorismate, a precursor in the synthesis of aromatic amino acids, ubiquinone, and vitamin K [16,17]. This route has been considered an excellent target for antimicrobials, given that it is only present in plants, bacteria, and apicomplexan parasites, being absent in humans [18].

The fifth step of the shikimate pathway is performed by shikimate kinase (SK). This enzyme catalyzes the phosphorylation of shikimate (SHK) to produce shikimate-3-phosphate (S3P), using ATP as a phosphate donor. SK is a member of the Nucleoside Monophosphate Kinase (NMP) family, a group of enzymes that are highly known by their major conformational changes during catalysis [19,20]. The structural characterization of SK from different bacteria showed that the enzyme is composed of three domains [21,22,23,24]: the core domain, which is formed by five β-sheets and a highly conserved phosphate-binding loop; the LID domain, which contains important residues for ATP binding and undergoes substantial structural changes upon substrate binding [25,26]; and the SHK-binding domain, which is responsible for the binding of shikimate [20].

In the search for new bioactive molecules, the benzimidazole nucleus has been long considered as a promising scaffold for the design of different type of drugs. Benzimidazole has shown its great versatility across diverse research areas, such as the design of antiviral, antifungal, antioxidant, and anticancer drugs [27], demonstrating its specific importance as an antimicrobial [28]. There have been previous reports of benzimidazole molecules tested against *S. aureus* [29,30], but with no molecular target specifically known.

In the present study, in vitro assays were performed with an in-house library of 170 benzimidazole derivatives against methicillin-resistant *Staphylococcus aureus* shikimate kinase (SaSK). Enzymatic inhibition assays were performed initially to identify hit compounds. Thereafter, the most potent were characterized to determine their inhibition mode. A structural characterization of the SaSK–inhibitor complex was performed through molecular dynamics simulation studies. Furthermore, their ADMETox properties were predicted by in silico tools. Finally, studies that included mitochondrial toxicity and antibacterial activity against an MRSA strain were also performed.

## 2. Results and Discussion

### 2.1. Inhibition Screening Assays

In the search for the first SaSK inhibitors, an in-house chemical library of 170 benzimidazole derivatives was screened against SaSK activity. Initially, the assays were performed at a concentration of 200 μM, but given that some of the compounds were not soluble under this condition, their highest possible concentration at the assay condition was employed. The results showed that out of the 170 molecules assessed, 10 compounds were able to inhibit SaSK by more than 30%, while 12 inhibited it between 20–29%, 77 performed an inhibition in the range of 10–19%, and the rest exhibited under 9% inhibition or showed no observable inhibition (Table S1). The chemical structure and assayed concentration from the 10 benzimidazole derivatives with the highest inhibition percentage are shown in Table 1. The most potent derivatives (**C1** and **C2**) inhibited the enzyme by 50 and 60%, at 70 and 100 µM, respectively.

It is noteworthy that even though all the compounds possess a benzimidazole nucleus, the percentage of inhibition depends on the functional groups as substituents. For example, a methyl group in position 1 has a detrimental effect (**C1** vs. **C4**). However, if a trifluoromethyl is added at position 2, the potency is recovered (**C2** vs. **C4**), but keeping this substituent alone it is not sufficient to increase the inhibition capability (**C2** vs. **C3** vs. **C4**). Furthermore, the length of the linker in position 5(6) induces an important increase in SaSK inhibition (**C2** vs. **C7**). Finally, it seems that the size of the substituent at position 5(6) in the benzimidazole nucleus plays a crucial role for potency in this type of molecules. As can be seen, compounds with the largest or bulky substituent (**C1**–**C4**) showed the highest inhibition capability, whilst those with a shorter substituent (**C8**–**C10**) showed the lowest inhibition capability. Therefore, compounds **C1** and **C2** represent the best combination for SaSK inhibition.

As it has been mentioned before, the benzimidazole nucleus has long been an attractive scaffold for the design of promising compounds with therapeutic effects, in particular, as antimicrobial agents. Owing to the immense importance and varied bioactivities exhibited by benzimidazole derivatives, the antibacterial activity of the azole class of compounds has been previously reported in literature [30,31,32,33].

### 2.2. Inhibition Mode

**Figure 1:**
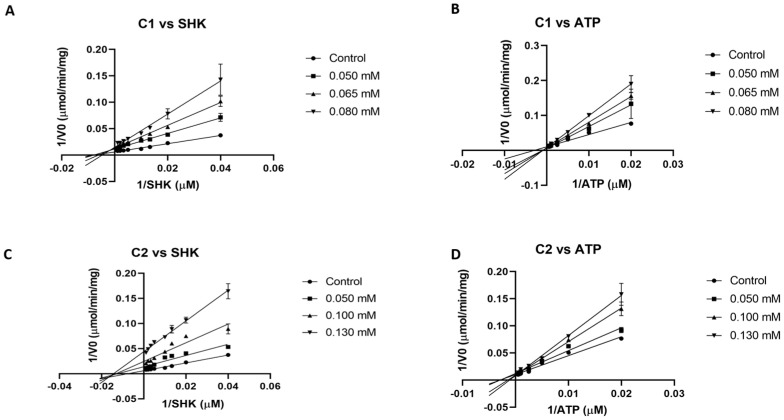
Lineweaver–Burk plots of SaSK inhibition. (**A**) Mode of inhibition **C1** vs. SHK. (**B**) Mode of inhibition **C1** vs. ATP. (**C**) Mode of inhibition **C2** vs. SHK. (**D**) Mode of inhibition **C2** vs. ATP.

The two most potent inhibitors (**C1** and **C2**) were evaluated to obtain their mode of inhibition with respect to both substrates (shikimate and ATP). First, due to solubility issues of compounds in assay conditions, a complete curve to calculate the IC50 value for both inhibitors could not be generated. Therefore, an estimated IC50 value of 70 and around 100 µM can be obtained from assays at fixed concentration for **C1** and **C2**, respectively (Table 1). From double reciprocal plots (Lineweaver–Burk plot), at different fixed inhibitor concentrations while varying one substrate and maintaining the other constant, both **C1** and **C2** were found to be competitive against ATP and non-competitive against shikimate, respectively (Figure 1). According to these data, a Ki value of 48 and 64 μM for SHK and ATP were calculated for **C1**. In the case of **C2,** these values were 50 and 101 μM, respectively.

It is well known that the ATP binding site of many enzymes is structurally related. This can complicate their development as therapeutic drugs; however, more and more strategies are emerging to be able to differentiate and take advantage of the small structural differences in this binding site [34]. There already exist reports that kinases are successful drug targets [35], where inhibitors compete with ATP for binding to the protein [36].

Regarding other shikimate kinases, several inhibitors have been reported previously, primarily for *M. tuberculosis* SK (MtSK), which include benzothiazole derivatives [37], aminobenzothiazole derivatives [38], natural products isolated from marine sponges, such as manzamine alkaloids and ilimaquinone [39,40], some synthetic compounds [41], naphthalene derivatives [42], and pyrazolone derivatives [43]. In the case of *H. pylori*, some synthetic inhibitors have been reported too [41,42,44]. It is important to mention that none of the compounds reported for other SKs have been assessed in SaSK. Therefore, the compounds reported here represent the first inhibitors found for this enzyme, becoming a good starting point in the search for new anti MRSA agents.

### 2.3. Docking and Molecular Dynamics Simulations

**Figure 2:**
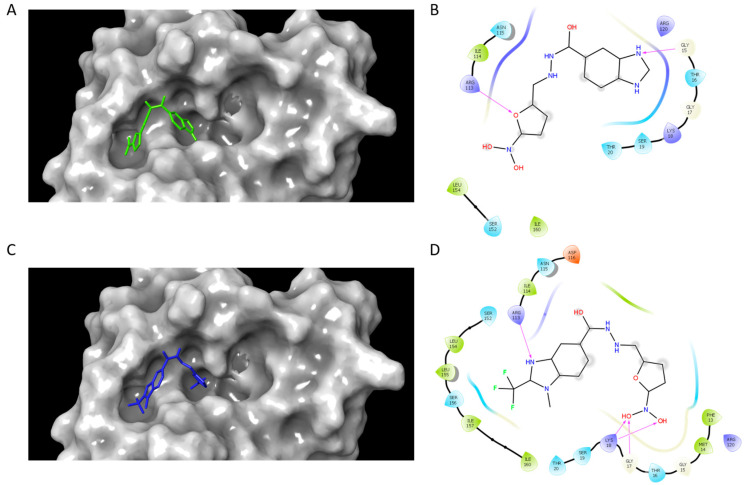
SaSK–inhibitor complex interactions. Binding pose and 2D interaction map at the ATP binding site of SaSK for **C1** (**A**,**B**) and **C2** (**C**,**D**). Protein is shown in molecular surface representation (**A**,**C**). Hydrogen bonds are depicted as arrows.

Once the inhibition mode was determined, it was important to obtain structural information related to protein–ligand interactions. To this end, a blind docking protocol was applied to determine the potential binding site of compounds **C1** and **C2**. The results showed that both compounds bound in the region corresponding to the ATP binding site and made a hydrogen bond interaction with ARG113. In homologous enzymes, the guanidine group of the corresponding arginine has been reported to be an essential residue for ATP binding and complex stabilization [25,26], given that it is in the LID region. Additionally, a hydrogen bond between GLY15 and **C1** was formed, whilst **C2** formed a hydrogen bond with GLY17 and LYS18 (Figure 2). ATP binding sites are known to have regions with conserved amino acids which include the Walker A motif (a loop with the sequence [A/G]xxxxGK[S/T], where x stands for any amino acid) and the Walker B motif (hhhhD[D/E], where h stands for any hydrophobic residue) [45]. It has been reported that in the catalytic domain, there is a region rich in glycine residues near an important lysine residue (LYS18), which has been shown to be involved in ATP binding [46,47].

**Figure 3:**
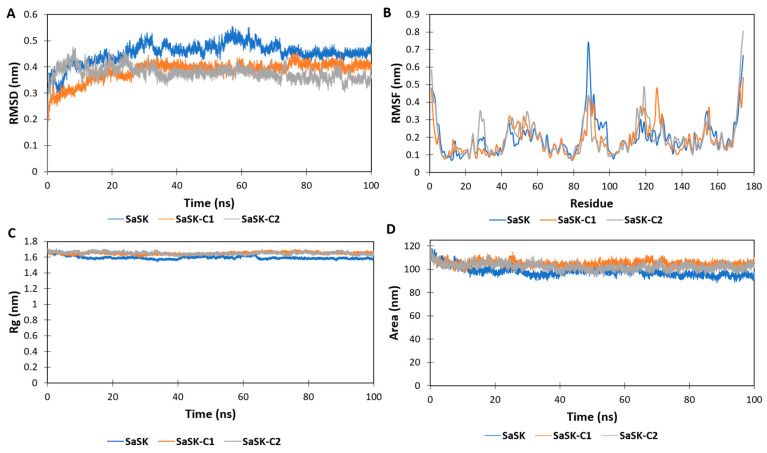
SaSK–inhibitor complex MD analysis. (**A**) RMSD, (**B**) RMSF, (**C**) Rg and (**D**) SASA along the 100 ns of simulated time.

In the MD simulations, it is possible to estimate how distinct parts of a molecule fluctuate at equilibrium and experience structural dynamic differences through a period [48]. Therefore, MD simulations were carried out to further characterize the dynamic behavior of the two compounds in complex with SaSK. The data showed that, according to root mean square deviation (RMSD) plots, both complexes were stable during the simulation time after a period of stabilization during the first 20 ns (Figure 3A). On the other hand, the root mean square fluctuations (RMSF) plot demonstrated important fluctuations in the ATP binding site, the LID region, and the CORE domain (Figure 3B). The LID region contains important residues for the stabilization of ATP binding. It is of notice that ARG113 and ARG120 are both in this region. In particular, ARG120 participates in phosphate transfer. The high mobility of this region is consistent with what is reported in other shikimate kinases [49] and NMP-Type enzymes in general, where both the LID and ATP binding regions are proven to be extremely mobile [21,22,23,24,25]. The radius of gyration (RG) and solvent accessible surface area (SASA) plots indicated that in both complexes, the protein remained in a compact state, which further confirms the stability of the binding (Figure 3C,D). With respect to the inhibitors reported for other SKs, an interaction with ARG113 (SaSK numbering) was observed, suggesting that this residue is important for the inhibition of this type of enzyme.

### 2.4. Minimum Inhibitory Concentration (MIC) in MRSA

**Figure 4:**
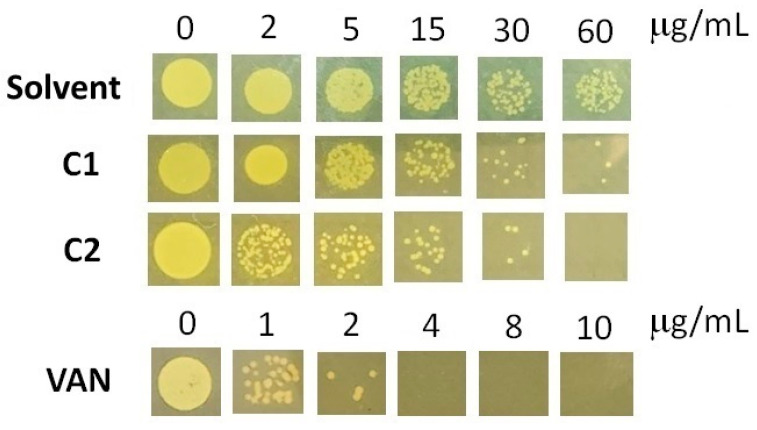
MIC determination for MRSA against **C1**, **C2**, and vancomycin. The images correspond to plates taken at 24 h. The experiments were performed in triplicate, the data shown are a representative image.

Benzimidazole and bis-benzimidazole derivatives were shown to have a strong ability to inhibit MRSA strains when tested. Benzimidazoles act as DNA-binding agents, antibiofilm agents, and enzyme inhibitors, showing a synergistic effect with antibiotics [50]. In this study, the MIC values for the **C1** and **C2** compounds were determined. MIC values are defined as the lowest concentration of each compound that was able to inhibit visible growth in agar plates. Our compounds showed moderate anti-MRSA activity. The MIC for **C1** was >60 μg/mL while **C2** had a MIC of 60 μg/mL (Figure 4). Even though **C1** was more effective than **C2**, it had a higher MIC value than those previously reported [51,52]. In these studies, a compound was found to have potent anti-MRSA activity (MIC 4 µg/mL). Therefore, it would be advantageous to examine the structural relationships between these compounds and those tested in this study and enhance our molecules. In addition, assays with vancomycin as a positive control showed that our MRSA strain can be considered as vancomycin-intermediate (MIC 4 µg/mL, Figure 4), according to the CLSI’s official interpretation, which considers an MIC of 2 µg/mL for MRSA susceptible to vancomycin [53]).

### 2.5. ADMETox Predictions

ADMETox characteristics have become important in the characterization of potential drug molecules before clinical trials, as highly toxic and poor pharmacokinetics could become a liability and ruin the expensive phases of drug design. According to the predictions by DataWarrior software [54], the results showed that neither **C1** nor **C2** presented adverse characteristics in the tumorigenic, mutagenic, irritating effects, or reproductive effects parameters. Moreover, their predicted pharmacokinetic characteristics using PreADMET software [55] suggested that both compounds appear to possess good chemical qualities and characteristics to potentially become a drug (Table 2). However, it should be noted that the in silico toxicological analysis using the online servers admetSAR and ADMETlab [56,57] has shown that both compounds have the potential to cause damage to cell organelles. For example, both compounds showed a predicted toxic effect on mitochondria, affecting their membrane potential either by inhibiting the respiratory chain or damaging mitochondrial membranes. These results agree with those reported in the Mitotox database, which reports four results with different benzimidazole derivatives (https://www.mitotox.org/, accessed on 28 February 2024).

### 2.6. Mitochondrial Toxicity Assays

**Figure 5:**
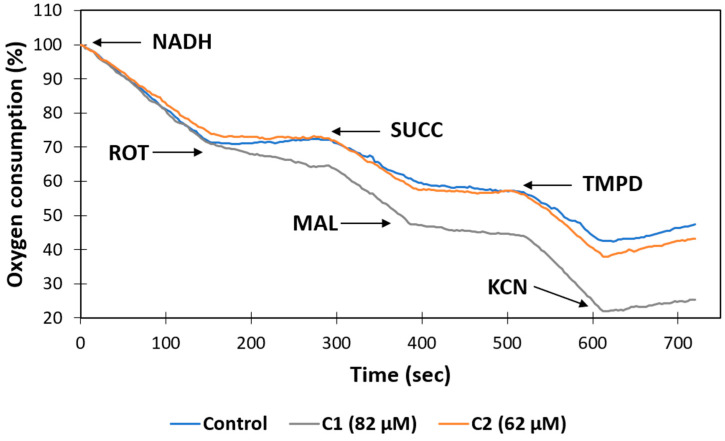
Effects of **C1** and **C2** on resting mitochondrial oxygen consumption (MOC, State 4) in isolated rat liver mitochondria. MOC was monitored in the presence of 1% DMSO or EtOH (vehicles, blue) or the indicated concentration of compounds (orange and gray).

Many drugs and toxins can cause side effects associated with mitochondrial damage, a phenomenon that has lately gained biological relevance. Drugs can affect mitochondrial function by a variety of mechanisms [58]. One well-defined mechanism is the inhibition of the electron transfer chain (complex I–IV) [59,60,61]. Therefore, to corroborate the in silico data about the potential mitochondrial toxicity of the compounds **C1** and **C2** predicted by the admetSAR and ADMETlab servers, we chose to test their effects on respiration in mitochondria isolated in the presence of different substrates and inhibitors of complex I, II, and IV. To conduct mitochondrial toxicity experiments, male rat liver mitochondria were extracted and treated with 10 µL of DMSO or **C1** and **C2** during 30 min. At the concentration of 62 µM, compound **C2** caused a slight decrease of 14% in respiration by the mitochondrial complexes (CI-III-IV), using nicotinamide adenine dinucleotide reduced form (NADH) as a substrate, while compound **C1**, at a concentration of 82 µM, only affected the consumption of oxygen through CI-III-IV in a non-significant way. However, it is important to mention that only the presence of **C1** caused a resistance of 25% to inhibition by rotenone (Figure 5), which suggests that this compound may interact with the quinone site in complex I. This result is somewhat consistent with reports who showed that 3 nM of benzimidazole inhibited the activity of complex I [62]. In addition, 6-nitrobenzimidazole decreases mitochondrial membrane potential in qHTS-HepG2 cells [63], and 2-[(E)-2-(4-Chlorophenyl)ethenyl]-1-methyl-1H-benzimidazole inhibits mitochondrial *sn*-glycerol 3-phosphate dehydrogenase in skeletal muscle mitochondria [64].

On the other hand, the respiration of the complexes (CII-CIII-CIV) with succinate (SUCC) was stimulated by the presence of both compounds by 22% (**C1**) and 14% (**C2**) and favored the inhibitory effect of malonate (MAL) (Figure 5). There is no literature on the effect of benzimidazole derivates on mammalian succinate dehydrogenase, and only a few studies reported that benzimidazole inhibits the parasite and fungi complex II [65,66]. Furthermore, it is important to mention that there is no literature on the effect of benzimidazole derivatives that modify the sensitivity of mitochondrial complexes to classic inhibitors such as rotenone and malonate. Therefore, additional trials will be necessary to understand the relevance of this information.

Finally, the activity of complex IV was evaluated using N,N,N′,N′-tetramethyl-p-phenylenediamine (TMPD) and ascorbate to promote the reduction of cytochrome C. Both compounds presented a slight activating effect on the activity of complex IV. Furthermore, the sensitivity of CIV to cyanide was not affected (Figure 5). These results agree with data reported elsewhere [64].

## 3. Materials and Methods

### 3.1. Protein Purification

SaSK was purified following a modified version of the method reported by Favela, et al. [67]. Briefly, *E. coli BL21* strain transformed with the plasmid that contains *aroK* gen was grown in 250 mL of LB broth supplemented with kanamycin (50 µg/mL). Overexpression was performed by inducing the culture when it reached 0.75 OD with 0.5 mM isopropyl β-D-thiogalactopyranoside (IPTG) at 20 °C during 4 h. Thereafter, cells were harvested, resuspended in lysis buffer, and sonicated while maintaining cold conditions. The lysate was centrifugated to separate the soluble fraction from the cell debris, and the supernatant was loaded onto a Ni-nitriloacetic acid affinity column previously equilibrated with washing buffer. SaSK was eluted by using a 400 mM imidazole concentration. Protein purification was confirmed by 15% SDS-PAGE.

### 3.2. Inhibition Screening Assays

The activity of purified SaSK was measured by a coupled assay described by Favela et al., [67] following the oxidation of NADH to NAD, monitored at 340 nm using an Hp Agilent 8453 UV-VIS spectrophotometer. The assay mixture contained 100 mM Tris-HCl pH 7.6, 5 mM MgCl_2_, 100 mM KCl, 2 mM ATP, 1 mM shikimate, 1 mM phosphoenolpyruvate, 6 μg/mL pyruvate kinase, 6 μg/mL lactate dehydrogenase, 0.25 mM NADH, 10% of dimethyl sulfoxide (DMSO), and 70 ng of purified SaSK protein. All assays were performed in triplicate at 25 °C. It is important to mention that at the percentage of DMSO used, the activity of the enzyme was not affected.

The primary screening of the benzimidazole compound library was carried out at a fixed concentration of 200 µM or at the maximum solubility possible for compounds with poor solubility in assay conditions. All the compounds were dissolved in DMSO.

### 3.3. Evaluation of Inhibition Mode

The inhibition mode of the compounds with the highest inhibition capacity was analyzed by measuring the effect of inhibitor concentration on enzymatic velocity as a function of substrate concentration. First, different concentrations of shikimate (0.025–1 mM) were employed, maintaining the ATP concentration fixed (2 mM) in the presence of varied concentrations of the selected compound. Next, the ATP concentration was varied (0.05–2 mM), keeping the shikimate concentration constant (1 mM) and varying concentrations of the selected compound. The obtained data (mean ± SD) were analyzed in GraphPad Prism 8 (GraphPad Software, Inc., La Jolla, CA, USA) software to obtain the inhibition mode and Ki value.

### 3.4. Docking and Molecular Dynamics Simulations Studies

To analyze the interactions of the selected compounds with SaSK, docking and molecular dynamics simulations were performed. The 3D homology model of SaSK reported previously was employed [67]. Prior to docking, the protein structure was prepared using Autodock Tools software [68], involving the addition of hydrogens, partial charges, and energy minimization of the structure. In order to allow nonbiased results, blind docking studies were carried out by placing the grid box around the entire protein structure. Docking simulations were performed using Autodock Vina docking software [69].

The 2D structures of compounds were sketched using Chemsketch software V12.01 [70]. Afterwards, the 3D structures of the selected compounds were constructed and prepared for docking with prepare_ligand4.py module [68].

Molecular dynamics simulations were carried out in GROMACS v5.1.5 [71] using the GROMOS54a7 force field. Prior to the simulation, the structure of each inhibitor was parameterized in the PRODRG server [72], obtaining the topology of each inhibitor, which was subsequently fused to form the SaSK–inhibitor complexes. Additionally, a control simulation was carried out with the apoenzyme. Each system was immersed in the center of a dodecahedral box, where the system was solvated by adding water using the simple point charge model (SPC) [73], and Na^+^ and Cl^−^ ions were added to neutralize the system.

An energy minimization simulation was carried out with a time lapse of 100 ps to reach a local minimum and reduce the energy of the system. Then, it was subjected to temperature and pressure equilibrium by performing two steps of 100 ps and 500 ps, respectively, using an isothermal-isochoric assembly (NVT), followed by an isothermal-isobaric assembly (NPT), bringing the system to a stable temperature and pressure. Finally, molecular dynamics simulations of each complex were carried out over a time scale of 100 ns, as well as for the apoenzyme. Subsequently, the data were analyzed using Visual Molecular Dynamics (VMD) software [74]. Further analyses were carried out by calculating the root mean square deviation (RMSD), the root mean square fluctuation (RMSF), the radius of gyration (Rg), and solvent accessible surface area (SASA).

### 3.5. Physicochemical and Toxicological Properties

In order to further characterize the inhibitors, the chemical structures of each of them were analyzed in silico in Data Warrior 4.07.02 software [54] and in the Pre-ADMET server [55]. Both platforms were used to obtain important data on each of the inhibitors, such as “drug-like” properties or properties related to the pharmacokinetics and pharmacodynamics of the inhibitors, such as absorption in the human intestine, binding to plasma proteins, penetration of the blood–brain barrier, inhibition of CYP450, and inhibition of hERG, among others. In addition, the ADMETlab 2.0 and admetSAR 3.0 servers were used to determine mitochondrial toxicology [56,57].

### 3.6. Minimum Inhibitory Concentration Assays

The MRSA252 strain (ATCC^®^ BAA-1720TM, ATCC^®^, Manassas, VA, USA) was used for the assays. The minimum inhibitory concentration (MIC) was calculated using the broth microdilution technique, as per the Clinical and Laboratory Standard Institute’s (CLSI) recommendations for aerobic bacteria, but with Mueller Hinton agar plate to promote its growth. The **C1** and **C2** compounds (2 to 60 µg/mL) were tested in agar plates with an initial inoculum of 5 × 10^5^ CFU/mL. Microtiter trays were incubated at 37 °C for 24 h under aerobic conditions. The negative control was one that was free of compounds and contained only the bacterial suspension and broth. A control curve including vancomycin was also performed as a positive control. The MIC was defined as the lowest concentration producing no visible growth.

### 3.7. Mitochondrial Toxicity Assays

#### 3.7.1. Mitochondrial Isolation

Mitochondria extraction was carried out by maintaining cold conditions. Male liver tissue was placed in approximately 200 mL of saline and three washes were performed to remove excess blood. The tissue was finely minced and homogenized with oximetry medium plus BSA in a Potter-Elvehjem-type tissue homogenizer. The resulting homogenate was centrifuged at 5000 rpm for 10 min at 4 °C; subsequently, the supernatant obtained was centrifuged at 12,000 rpm for 10 min at 4 °C. Finally, the pellet was resuspended in oximetry medium that contained 10 mM KH_2_PO_4_, 250 mM sucrose, 2.5 mM MgCl, and 5 mM EDTA at a pH of 7.2. The mitochondria were kept on ice to be used in subsequent assays.

#### 3.7.2. Oxygen Uptake

Oxygen uptake was estimated using a Clark-type electrode in a 0.6 mL chamber at 25° C. Oximetry medium that contained 10 mM KH_2_PO_4_, 250 mM sucrose, 2.5 mM MgCl, and 5 mM EDTA at a pH 7.2 was used. Oxygen consumption was stimulated by the addition of 2.5 mM NADH or 7.1 mM succinate (in the presence of 3 μM rotenone). Artificial substrates such as TMPD (0.77 mM) were used for complex IV activity, and malonate and KCN were added to inhibit complex II and complex IV (1.42 mM and 0.85 mM, respectively).

### 3.8. Synthesis of Benzimidazole Derivatives (C1–C10)

#### 3.8.1. Instrumental Analysis

To measure the range of melting points, the Büchi Melting Point B—540 apparatus was used.

Infrared spectroscopy (IR): spectra were recorded using a Perkin-Elmer Spectrum 400 FTIR/FIR, employing fully attenuated reflectance ATR, with a working range of 4000–400 cm^−1^.

Nuclear magnetic resonance spectroscopy (NMR): spectra were recorded using a Varian VNMRS (^1^H = 400 MHz, ^13^C = 101 MHz) apparatus. Chemical shifts (δ) are reported in parts per million (ppm) relative to tetramethylsilane (TMS), DMSO, with coupling constants (*J*) expressed in Hz. The following abbreviations are used for multiplicities: s = singlet; d = doublet; dd = doublet of doublets; t = triplet; q = quartet; m = multiplet.

High-resolution mass spectrometry (HRMS): spectra were recorded using a Perkin-Elmer AxION^®^ 2 TOF (time-of-flight) for atmospheric pressure chemical ionization (APCI-HRMS). The solvent acetonitrile from J. T. Baker was used, and the samples were introduced via direct sample analysis (DSA). Molecular weights were obtained to four decimal places.

High-pressure liquid chromatography-diode-array detector (HPLC-DAD): spectra were recorded using an Agilent Infinity 1260 with an Agilent column with the following properties: C18 extend, 5 µm, and dimensions of 4.6 × 10 mm. UV detection was performed using a photodiode array scanning from 190 to 600 nm. The elution mixture used was 8:2 water:acetonitrile, and the solvents were obtained from J. T. Baker.

#### 3.8.2. Synthesis

Compounds **C5** [75], **C6**, **C7** [76], **C8** [77], **C9** [78], and **C10** [79] were taken from our in house library, and their synthesis was reported previously.

**Figure 6:**
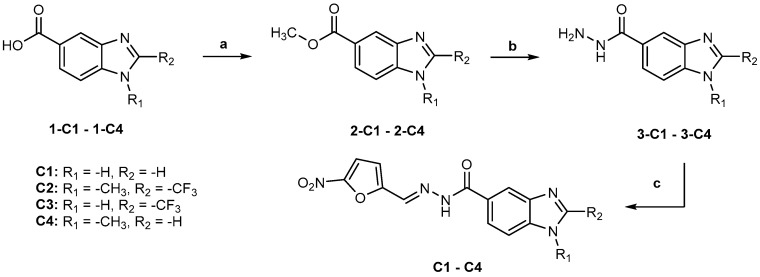
Synthesis of compounds **C1**–**C4**. Reactants and conditions: (**a**) H_2_SO_4_, MeOH, 70 °C; (**b**) NH_2_NH_2_:H_2_O, MeOH, reflux; (**c**) 5-Nitro-2-furaldehyde, AcOH, rt.

Compounds **C1**, **C2**, **C3**, and **C4** were prepared from properly substituted benzimidazol-5-carboxylic acids (**1-C1**–**1-C4**) by esterification, followed by the preparation of 1*H*-benzimidazole-5-carbohydrazides and *N′*-((5-nitrofuran-2-yl)methylene)-1*H*-benzoimidazole-5-carbohydrazides, as shown in Figure 6.

Synthesis of methyl esters (**2-C1**–**2-C4**).

The benzimidazol-5-carboxylic acids properly substituted were converted to methyl esters by Fisher’s method. Briefly, compounds **1-C1**–**1-C4**, were solved in MeOH anh. and treated with 2.2 eq. of H_2_SO_4_ at 65–70 °C for 72 h. After that, the solvent was removed, and the residue was neutralized with a saturated dissolution of NaHCO_3_. Esters were recuperated by EtOAc extractions, and the crude products were purified by MeOH recrystallization, resulting in amorphous powders.

Methyl 1*H*-benzimidazole-5-carboxylate (**2-C1**). Yield 75%, mp 123.4–127.8 °C. FTIR-ATR: 3095.64 (vN-H), 2810.90 (vCH3-O), 711.23 (vC=O), 1625.58 (vC=N). ^1^H NMR: (400 MHz, DMSO-*d_6_*) δ (ppm): 12.78 (s, 1H, D_2_O), 8.41 (s, 1H), 8.23 (d, *J* = 1.7 Hz, 1H), 7.84 (dd, *J* = 8.5, 1.6 Hz, 1H), 7.68 (d, *J* = 8.5, 0.6 Hz, 1H), 3.86 (s, 3H). ^13^C NMR: (101 MHz, DMSO-*d_6_*) δ (ppm): 166.84 (**C**=O), 163.10, 163.10, 144.66 (N-**C**=N), 123.26, 122.96, 117.62, 114.90, 51.96 (O-**C**H_3_).

Methyl 1-methyl-2-trifluoromethyl-1*H*-benzimidazole-5-carboxylate (**2-C2**). Yield 76%, mp 119.4–120.3 °C. FTIR-ATR: 2962.89 (v_CH3-O_), 2847.49 (v_CH3-N_), 1722.47 (v_C=O_), 1621.70 (v_C=N_) 1125.74 (v_CF3_). ^1^H NMR: (400 MHz, DMSO-*d_6_*) δ (ppm): 8.33 (s, 1H), 8.01 (dd, *J* = 8.8. 1.3 Hz, 1H), 7.84 (d, *J* = 8.7 Hz 1H), 3.98 (s, 3H), 3.88 (s, 3H). ^13^C NMR: (101 MHz, DMSO-*d_6_*) δ (ppm): 166.17 (**C**=O), 141.69 (q, *J* = 38.1 Hz, **C**-CF_3_), 139.84, 139.10, 125.44, 124.95, 122.39, 118.70 (q, **C**F_3_), 111.93, 52.17(O-**C**H_3_), 31.24 (N-**C**H_3_).

Methyl 2-trifluoromethyl-1*H*-benzimidazole-5-carboxylate (**2-C3**). Yield 87%, mp 160.2–161.9 °C. FTIR-ATR: 3237.57 (v_N-H_), 2958.37 (v_CH3-O_), 1695.59 (v_C=O_), 1625.58 (v_C=N_), 1133.63 (v_CF3_). ^1^H NMR: (400 MHz, DMSO-*d_6_*) δ (ppm): 14.39 (s, 1H, D_2_O), 8.30 (s, 1H), 7.95 (dd, *J* = 8.6. 1.6 Hz, 1H), 7.79 (dd, *J* = 8.6 Hz, 1H), 3.88 (s, 3H). ^13^C NMR: (101 MHz, DMSO-*d_6_*) δ (ppm): 166.27 (**C**=O), 142.40 (q, *J* = 39.6 Hz, **C**-CF_3_), 142.12, 138.85, 125.40, 124.89, 119,42 (q, *J* = 51.2 Hz, **C**F_3_), 118.84, 115.77, 52.20.

Methyl 1-methyl-1*H*-benzimidazole-5-carboxylate (**2-C4**). Yield 85%, mp 123.9–125.5 °C. FTIR-ATR: 2955.68 (v_CH3-O_), 2846.10 (v_CH3-N_), 1702.36 (v_C=O_), 1620.26 (v_C=N_). ^1^H NMR: (400 MHz, DMSO-*d_6_*) δ (ppm): 8.34 (s, 1H), 8.25 (d, *J* = 1.6 Hz, 1H), 7.89 (dd, *J* = 8.5, 1.5 Hz, 1H), 7.65 (d, *J* = 8.6 Hz, 1H), 3.87 (s, 3H), 3.86 (s, 3H). ^13^C NMR: (101 MHz, DMSO-*d_6_*) δ (ppm): 166.79 (**C**=O), 146.78 (N-**C**=N), 142.90, 137.89, 123.27, 123.08, 121.05, 110.33, 51.94 (O-**C**H_3_), 30.88 (N-**C** H_3_).

Synthesis of carbohydrazides (**3-C1**–**3-C4**)

The methyl benzimidazol-5-carboxylates **2-C1**–**2-C4** properly substituted were solved in MeOH and treated with 8–12 eq. of N_2_H_4_ solution 35 wt. % in H_2_O at reflux for 48 h. After that, the solvent was removed, and the residue was mixed with a brine solution and was stirred for 5 min at 5 °C or until a slurry was formed. The mixture was filtered and washed with cold water. Finally, the crude products were recrystallized from EtOH and were recuperated in the form of amorphous powders.

1*H*-benzimidazole-5-carbohydrazide (**3-C1**). Light orange powder. Yield 74%, mp 258.7–263.8 °C. ^1^H NMR: (600 MHz, DMSO-*d_6_*) δ (ppm): 12.74 (s, 1H, D_2_O), 9.76 (s, 1H, D_2_O), 8.34 (s, 1H), 8.12 (s, 1H), 7.73 (d, *J* = 8.3 Hz, 1H), 7.62 (s, 1H), 4.50 (s, 2H, D_2_O). ^13^C NMR: (151 MHz, DMSO-*d_6_*) δ (ppm): 166.70 (**C**=O), 143.81, 143.81, 127.14 (N-**C**=N), 121.51, 120.75, 118.22, 111.31.

1-Methyl-2-(trifluoromethyl)-1*H*-benzimidazole-5-carbohydrazide (**3-C2**). White powder. Yield 80%, mp 217.5–220.4 °C. ^1^H NMR: (600 MHz, DMSO-*d_6_*) δ (ppm): 9.88 (s, 1H, D_2_O), 8.30 (s, 1H), 7.98 (dd, *J* = 8.7, 1.9 Hz, 1H), 7.85 (d, *J* = 8.7 Hz, 1H) 4.53 (s, 2H, D_2_O), 3.99 (s. 3H). ^13^C NMR: (151 MHz, DMSO-*d_6_*) δ (ppm): 165.79 (**C**=O), 141.09 (q, *J* = 38.3 Hz, **C**-CF_3_), 139.85, 137.83, 128.89, 124.27, 119.67, 118.89 (q, *J* = 271.3 Hz, -**C**F_3_), 111.59, 31.20 (N-**C**H_3_).

2-(Trifluoromethyl)-1*H*-benzimidazole-5-carbohydrazide (**3-C3**). Light yellow powder. Yield 92%, mp 206.4–210.1 °C. ^1^H NMR: (400 MHz, DMSO-*d_6_*) δ (ppm): 9.70 (s, 1H, D_2_O), 8.13 (s, 1H), 7.68 (dd, *J* = 8.5, 1.7 Hz, 1H), 7.59 (dd, *J* = 8.5, 0.7 Hz, 1H), 4.84 (s, 2H, D_2_O). ^13^C NMR: (101 MHz, DMSO-*d_6_*) δ (ppm): 167.41 (**C**=O), 147.06 (q, *J* = 36.5 Hz, **C**-CF_3_), 143.83, 141.76, 126.97, 121.17, 120,98 (q, *J* = 270.7 Hz, **C**F_3_), 117.1, 116.66.

1-Methyl-1*H*-benzimidazole-5-carbohydrazide (**3-C4**). White powder. Yield 52%, mp 251.6–254.2 °C. ^1^H NMR: (600 MHz, DMSO-*d_6_*) δ (ppm): 9.74 (s, 1H, D_2_O), 8.27 (s, 1H), 8.16 (s, 1H), 7.80 (dd, *J* = 8.5, 1.6 Hz, 1H), 7.61 (d, *J* = 8.6 Hz, 1H) 4.47 (s, 2H, D_2_O), 3.85 (s. 3H). ^13^C NMR: (151 MHz, DMSO-*d_6_*) δ (ppm): 166.55 (**C**=O), 146.06 (N-**C**=N), 142.84, 136.47, 126.89, 121.64, 118.32, 109.94, 30.83 (N-**C** H_3_).

Synthesis of (methylene)-1H-benzimidazole-5-carbohydrazides (**C1**–**C4**).

The carbohydrazides **3-C1**–**3-C4** properly substituted were prepared by imine formation by reaction with 5-nitro-2-furaldehyde in acid conditions. Raw materials were solved in CHCl_3_ to equimolar quantities at room temperature. Acetic acid was added drop by drop until a clear solution was observed. After 12–18 h, the reaction mixture was cooled at 5 °C, then it was filtered and washed using cold CHCl_3_. Finally, the products were purified by two cold consecutive washes in CHCl_3_, stirring for 30 min to remove traces of unwanted compounds.

*N′*-[(5-Nitrofuran-2-yl)methylene]-1*H*-benzimidazole-5-carbohydrazide (**C1**). Intense canary powder. Yield 74%, mp 286.1–289.9 °C with decomposition. ^1^H NMR: (600 MHz, DMSO-*d_6_*) δ (ppm): 12.50 (s, 1H, D_2_O), 8.56 (s, 1H), 8.47 (s, 1H), 8.33 (s, 1H), 7.88 (d, *J* = 8.7 Hz, 1H), 7.79 (d, *J* = 3.8 Hz, 1H), 7.72 (d, *J* = 8.5 Hz, 1H), 7.26 (d, *J* = 3.9 Hz, 1H). ^13^C NMR: (151 MHz, DMSO-*d_6_*) δ (ppm): 163.87 (**C**=O), 152.09, 151.89, 144.32 (N-**C**=N), 139.78, 138.05, 135.05, 126.47, 122.31, 116.21, 115.02, 115.02, 114.79. HRMS for C_13_H_9_N_5_O_4_ [M^+^ + H] calcd. 300.07273, found 300.0704. HPLC-DAD: 97.1%.

1-Methyl-*N′*-[(5-nitrofuran-2-yl)methylene]-2-(trifluoromethyl)-1*H*-benzimidazole-5-carbohydrazide (**C2**). Light yellow powder. Yield 88%, mp 274.7–277.0 °C with decomposition. ^1^H NMR: (600 MHz, DMSO-*d_6_*) δ (ppm): 12.38 (s, 1H, D_2_O), 8.46 (s, 2H), 8.06 (d, *J* = 8.7 Hz, 1H), 7.97 (d, *J* = 8.6 Hz, 1H), 7.81 (d, *J* = 4.1 Hz, 1H), 7.29 (d, *J* = 4.0 Hz, 1H), 4.03 (s. 3H). ^13^C NMR: (151 MHz, DMSO-*d_6_*) δ (ppm): 163.21 (**C**=O), 151.96, 151.83, 141.67 (**C**-CF_3_), 139.79, 138.54, 135.46, 128.17, 124.99, 120.75, 118.84 (q, *J* = 270.9 Hz, -**C**F_3_), 115.38, 114.72, 112.14 31.36 (N-**C**H_3_). HRMS for C_15_H_10_F_3_N_5_O_4_ [M^+^ + H] calcd. 382.07577, found 382.0761. HPLC-DAD: 99.6%.

*N′*-[(5-Nitrofuran-2-yl)methylene]-2-(trifluoromethyl)-1*H*-benzimidazole-5-carbohydrazide (**C3**). Intense yellow powder. Yield 78%, mp 259.7–265.4 °C with decomposition. ^1^H NMR: (400 MHz, DMSO-*d_6_*) δ (ppm): 12.60 (s, 1H, D_2_O), 8.58 (s, 1H), 8.41 (s, 1H), 7.94 (d, *J* = 8.5, 1H), 7.79 (m, 2H), 7.27 (d, *J* = 4.0 Hz, 1H). ^13^C NMR: (151 MHz, DMSO-*d_6_*) δ (ppm): 163.72 (**C**=O), 152.05, 151.90, 144.02, 141.32, 139.35, 135.23, 127.47, 123.29, 119.46 (q, *J* = 270.7 Hz, **C**F_3_), 117.84, 116.28, 115.08, 114.77. HRMS for C_14_H_8_F_3_N_5_O_4_ [M^+^ + H] calcd. 368.06012, found 368.0614. HPLC-DAD: 97.5%.

1-Methyl-*N′*-[(5-nitrofuran-2-yl)methylene]-1*H*-benzimidazole-5-carbohydrazide (**C4**). Intense canary powder. Yield 95%, mp 296.0–299.7 °C with decomposition. ^1^H NMR: (600 MHz, DMSO-*d_6_*) δ (ppm): 12.22 (s, 1H, D_2_O), 8.40 (s, 1H), 8.30 (d, 2H), 7.90 (m, 1H), 7.77 (s, 1H), 7.68 (d, *J* = 7.8 Hz, 1H), 7.23 (s, 1H), 3.85 (s. 3H). ^13^C NMR: (151 MHz, DMSO-*d_6_*) δ (ppm): 163.76 (**C**=O), 152.02, 151.90, 146.70 (N-**C**=N), 142.77, 137.25, 134.92, 126.09, 122.51, 119.24, 115.08, 114.77, 110.52, 30.95 (N-**C**H_3_). HRMS for C_14_H_11_N_5_O_4_ [M^+^ + H] calcd. 314.08838, found 314.0915. HPLC-DAD: 99.2%.

## 4. Conclusions

The benzimidazole derivatives reported here are the first SaSK inhibitors, presenting a promising scaffold and serving as a guide for designing new drugs against MRSA. The two most potent compounds acted as competitive inhibitors against ATP and non-competitive inhibitors against SHK, interacting with residues involved in ATP binding. Biological activity assays revealed that they were able to inhibit MRSA growth, and mitochondrial toxicity experiments showed that both compounds were able to interact with electron transport chain complexes. Finally, ADMETox prediction studies suggested that both inhibitors have the potential to be considered as drug candidates.

## Figures and Tables

**Table 1:** Benzimidazole derivatives with the highest SaSK inhibition percentage.

Compound	Structure	Assessed Concentration (µM)	% Inhibition in SaSK
**(C1)**	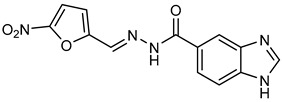	70	50
**(C2)**	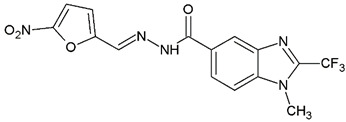	100	60
**(C3)**	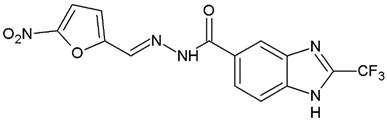	100	43
**(C4)**	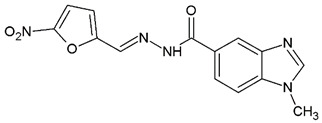	100	40
**(C5)**	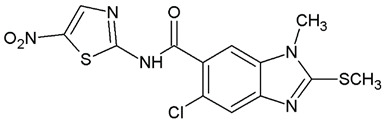	100	45
**(C6)**	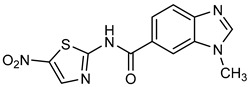	100	30
**(C7)**	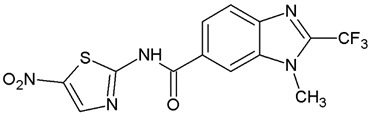	200	44
**(C8)**	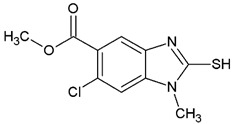	200	43
**(C9)**	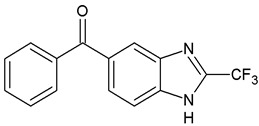	200	30
**(C10)**	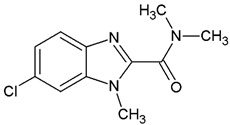	200	30

**Table 2:** Predicted pharmacokinetic properties from SaSK inhibitors.

	C1	C2
Blood–brain barrier	0.0391276	0.139873
In vitro permeability in Caco-2 cells (nm/s)	7.547	20.9485
Intestinal absorption (HIA, %)	76.777412	90.649752
In vitro permeability in MDCK cells (nm/s)	32.0734	9.12465
In vitro inhibition of P-glycoprotein	No	Yes
In vitro skin permeability (logKp, cm/h)	−4.58393	−2.41778
CYP 2C19 inhibition	No	No
CYP 2C9 inhibition	No	Yes
CYP 2D6 inhibition	No	No
CYP 2D6 substrate	No	No
CYP 3A4 inhibition	No	No
CYP 3A4 substrate	Si	Weak

Values were calculated with the PreADMET server. BBB: in vivo blood–brain barrier penetration (C.brain/C.blood), high absorption to CNS, more than 2.0; moderate absorption from 2.0 to 0.1; low absorption, less than 0.1; Caco–2: in vitro Caco−2 cell permeability (nm/s); values > 500 nm s^−1^ indicate good permeability and values < 25 nm s^−1^ indicate low permeability; HIA: human intestinal absorption (HIA, %); a high intestinal abortion percentage is desirable, as indicated by values closest to 100%; MDCK: in vitro MDCK (Mandin Darby Canine Kidney) cell permeability (nm/s); values > 500 nm s^−1^ indicate good permeability and values < 25 nm s^−1^ indicate low permeability; Pgp inhibition: in vitro P-glycoprotein inhibition; skin permeability: in vitro skin permeability–transdermal delivery (logKp, cm/h); CYP 2C19 inhibition: in vitro Cytochrome P450 2C19 inhibition; CYP 2C9 inhibition: in vitro Cytochrome P450 2C9 inhibition; CYP 2D6 inhibition: in vitro Cytochrome P450 2D6 inhibition; CYP 2D6 substrate: in vitro Cytochrome P450 2D6 substrate; CYP 3A4 inhibition: in vitro Cytochrome P450 3A4 inhibition; CYP 3A4 substrate: in vitro Cytochrome P450 3A4 substrate.

## Data Availability

The original contributions presented in the study are included in the article/Supplementary Material, further inquiries can be directed to the corresponding author/s.

## Supplementary Materials

The following supporting information can be downloaded at: https://www.mdpi.com/article/10.3390/ijms25105077/s1.
