# Supplementary material for: Inhibition of Shikimate Kinase from Methicillin-Resistant Staphylococcus aureus by Benzimidazole Derivatives. Kinetic, Computational, Toxicological, and Biological Activity Studies
Source: Int J Mol Sci. 2024 May 7;25(10):5077. doi: 10.3390/ijms25105077 (PMC11121535; doi:10.3390/ijms25105077)
Supplement: Supplementary file 1 [file ijms-25-05077-s001.zip › ijms-2942766-supplementary figures.pdf]

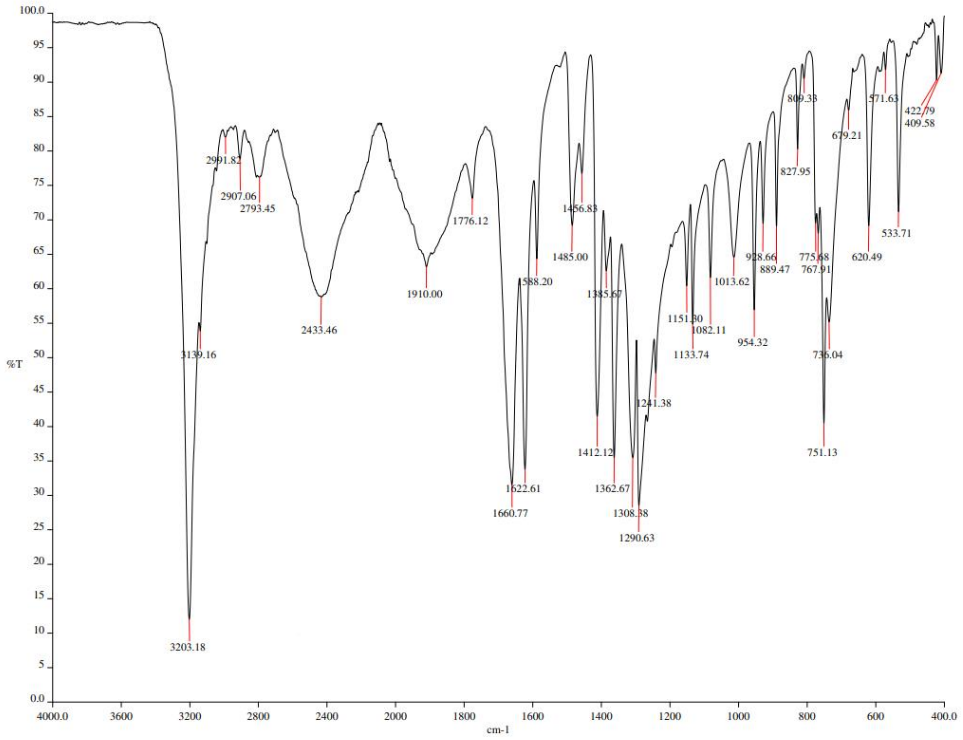

Figure 1. ATR-FTIR: 1*H*-Benzimidazole-5-carboxylic acid (**1-C1**).

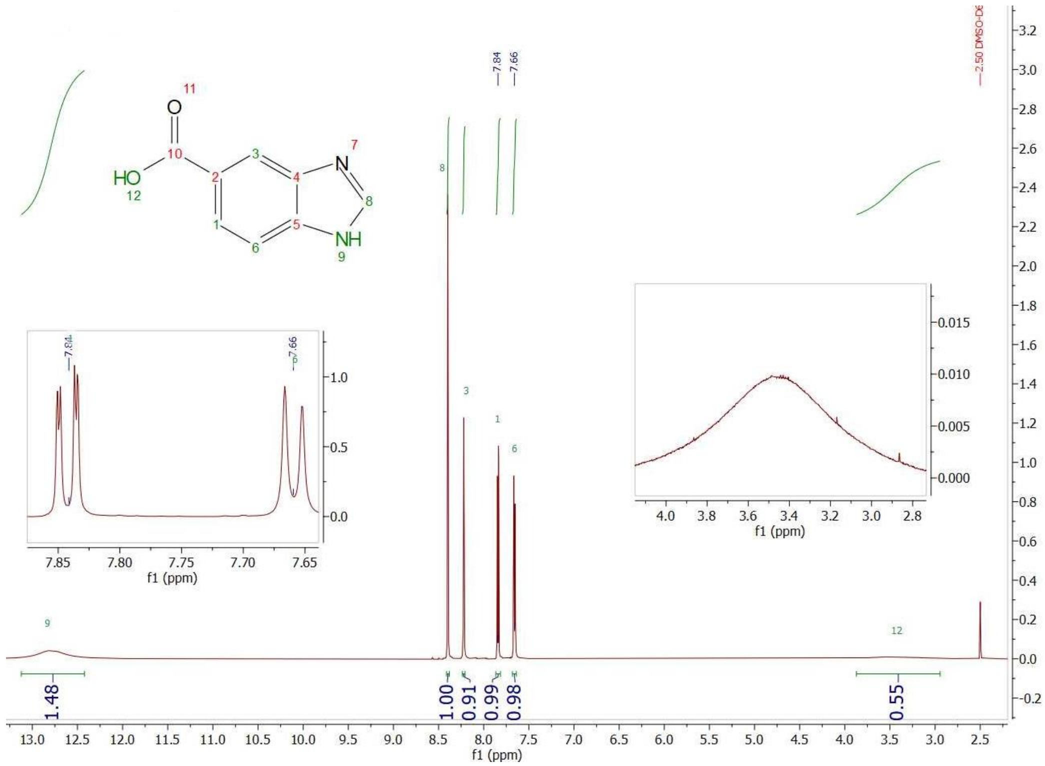

Figure 2. ^{1}H NMR (DMSO-d_6, 600 MHz): 1*H*-Benzimidazole-5-carboxylic acid (**1-C1**).

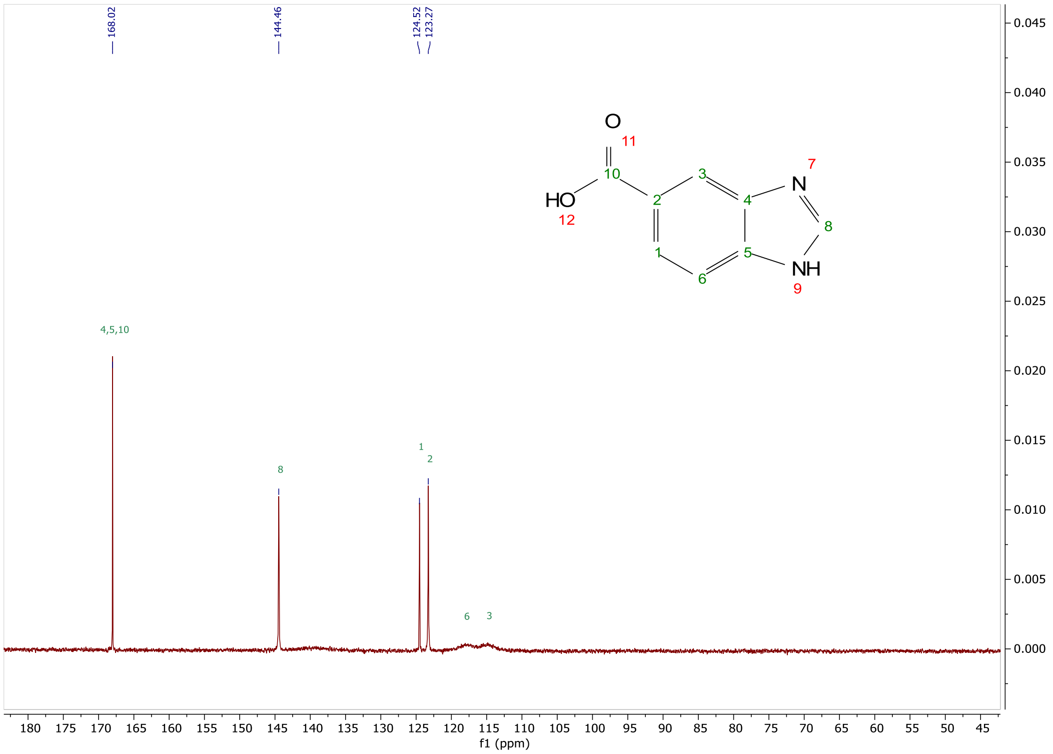

Figure 3. ^{13}C NMR (DMSO-d_6, 151 MHz): 1*H*-Benzimidazole-5-carboxylic acid (**1-C1**).

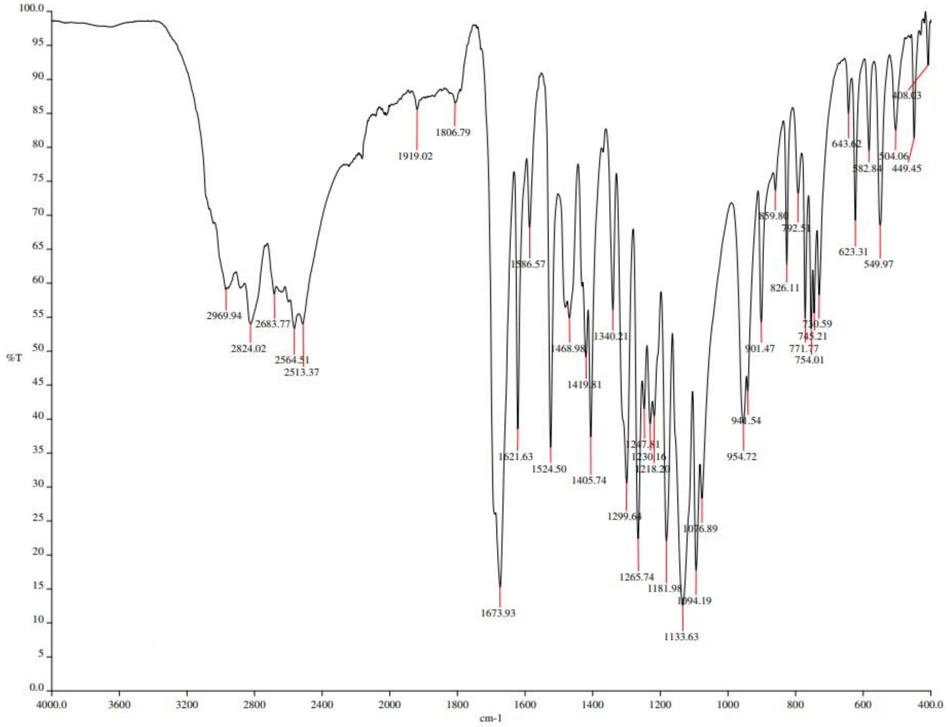

Figure 4. ATR-FTIR: 1-Methyl-2-trifluoromethyl-1*H*-benzimidazole-5-carboxylic acid (**1-C2**).

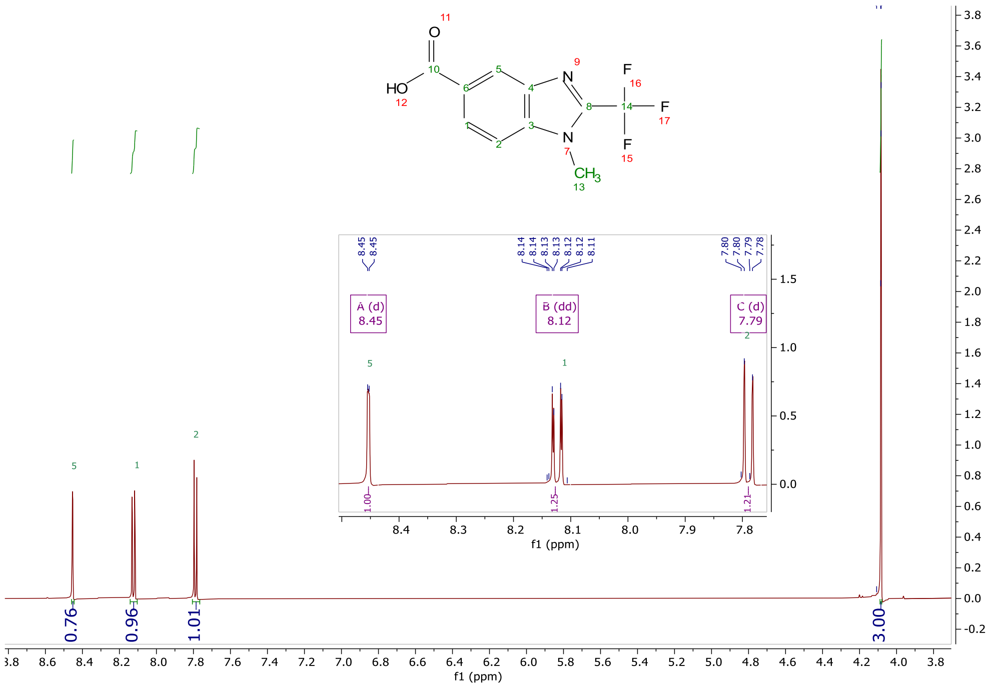

Figure 5. ^{1}H NMR (DMSO-d_6, 600 MHz): 1-Methyl-2-trifluoromethyl-1*H*-benzimidazole-5-carboxylic acid (**1-C2**).

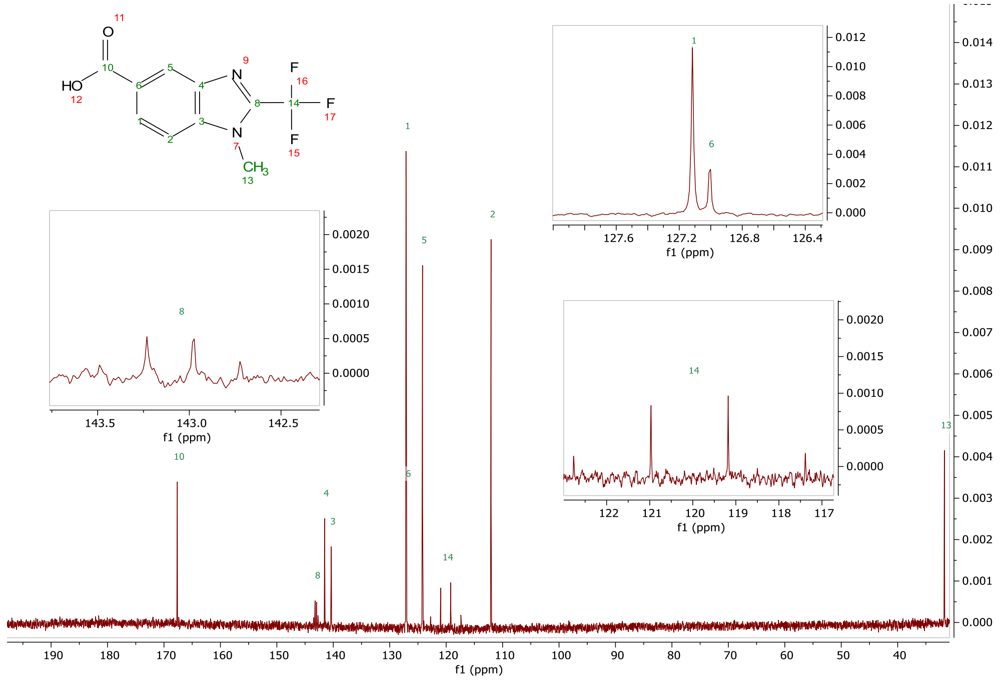

Figure 6. ^{13}C NMR (DMSO-d_6, 151 MHz): 1-Methyl-2-trifluoromethyl-1*H*-benzimidazole-5 carboxylic acid (**1-C2**).

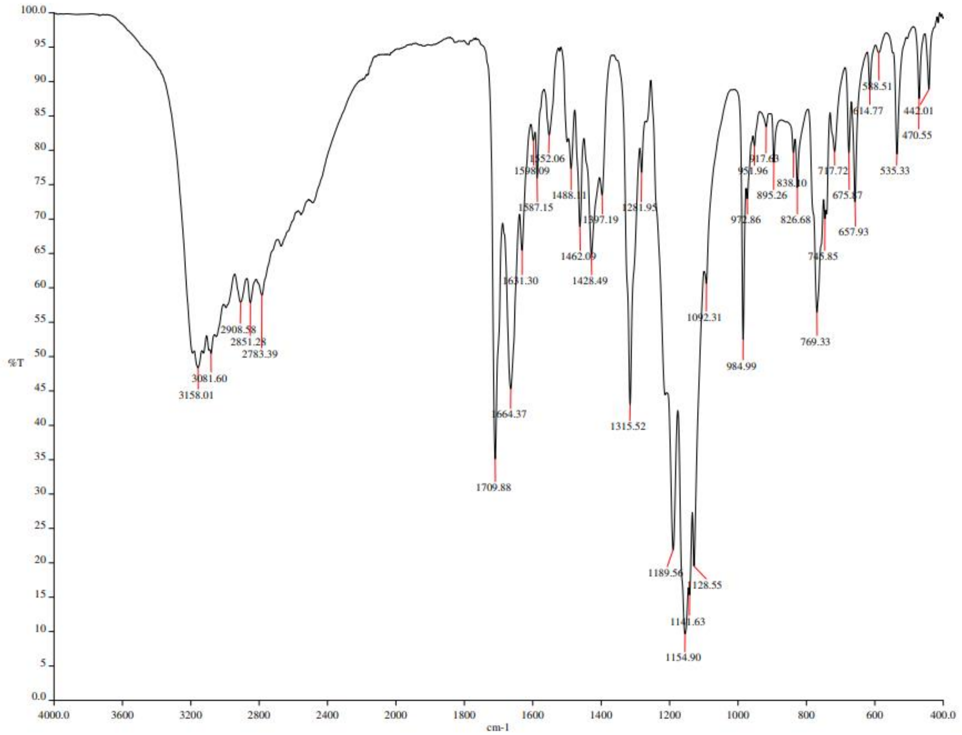

Figure 7. ATR-FTIR: 2-Trifluoromethyl-1*H*-benzimidazole-5-carboxylic acid (**1-C3**).

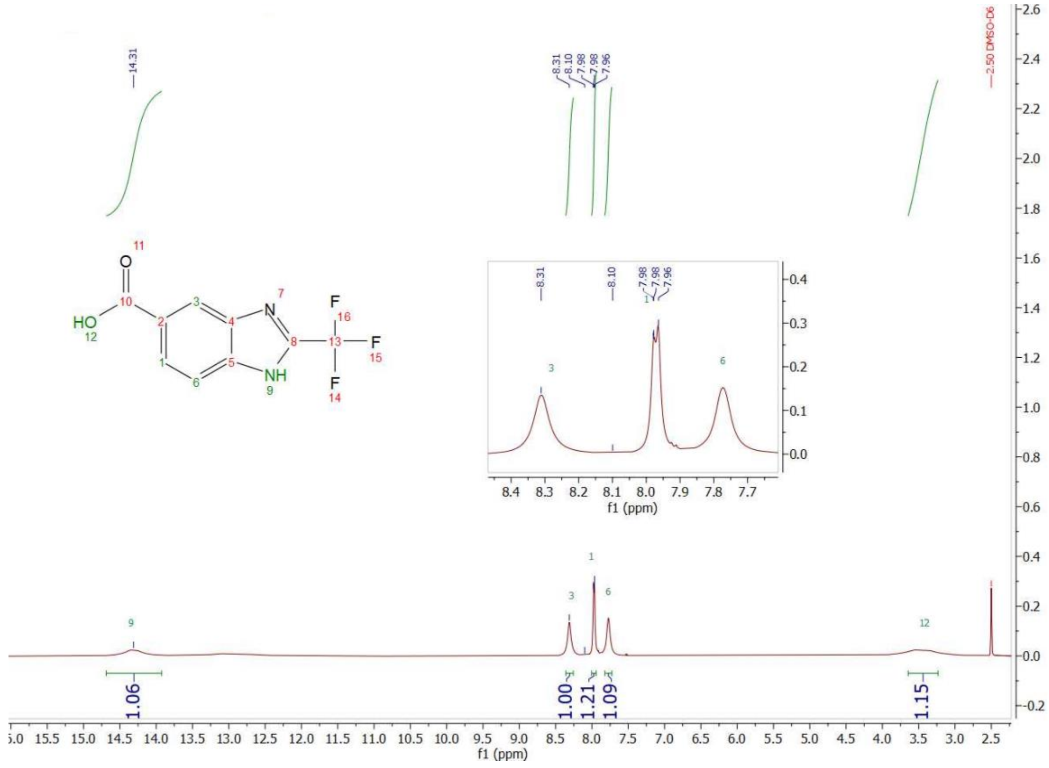

Figure 8. ^{1}H NMR (DMSO-d_6, 600 MHz): 2-Trifluoromethyl-1*H*-benzimidazole-5-carboxylic acid (**1-C3**).

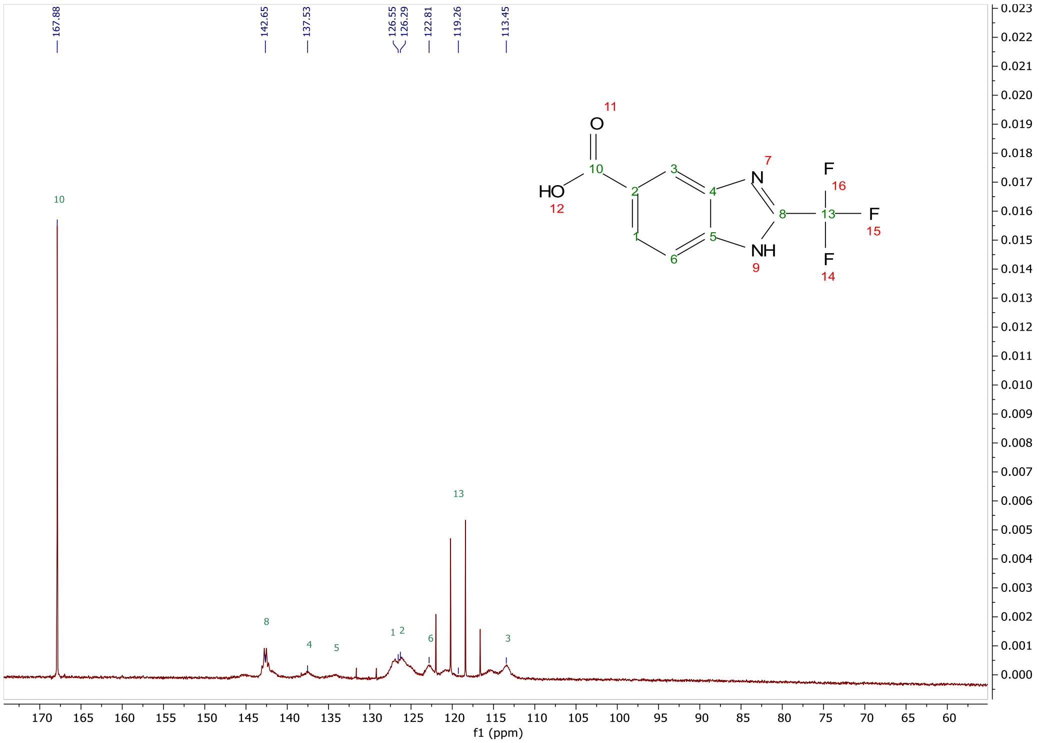

Figure 9. ^{13}C NMR (DMSO-d_6, 151 MHz): 2-Trifluoromethyl-1*H*-benzimidazole-5-carboxylic acid (**1-C3**).

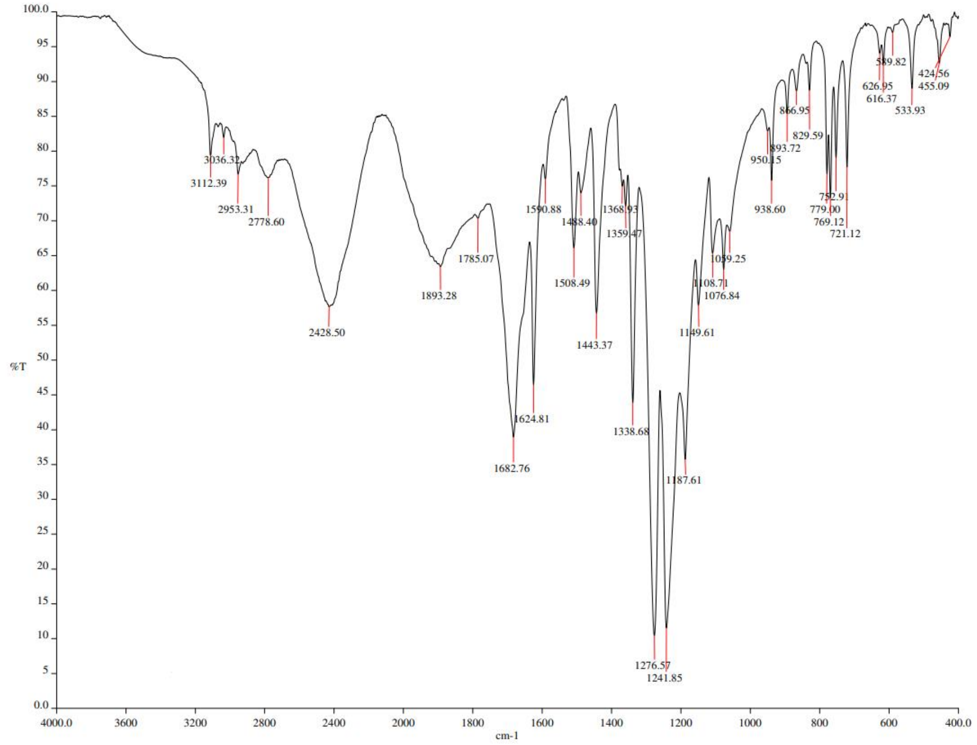

Figure 10. ATR-FTIR: 1-Methyl-1*H*-benzimidazole-5-carboxylic acid (**1-C4**).

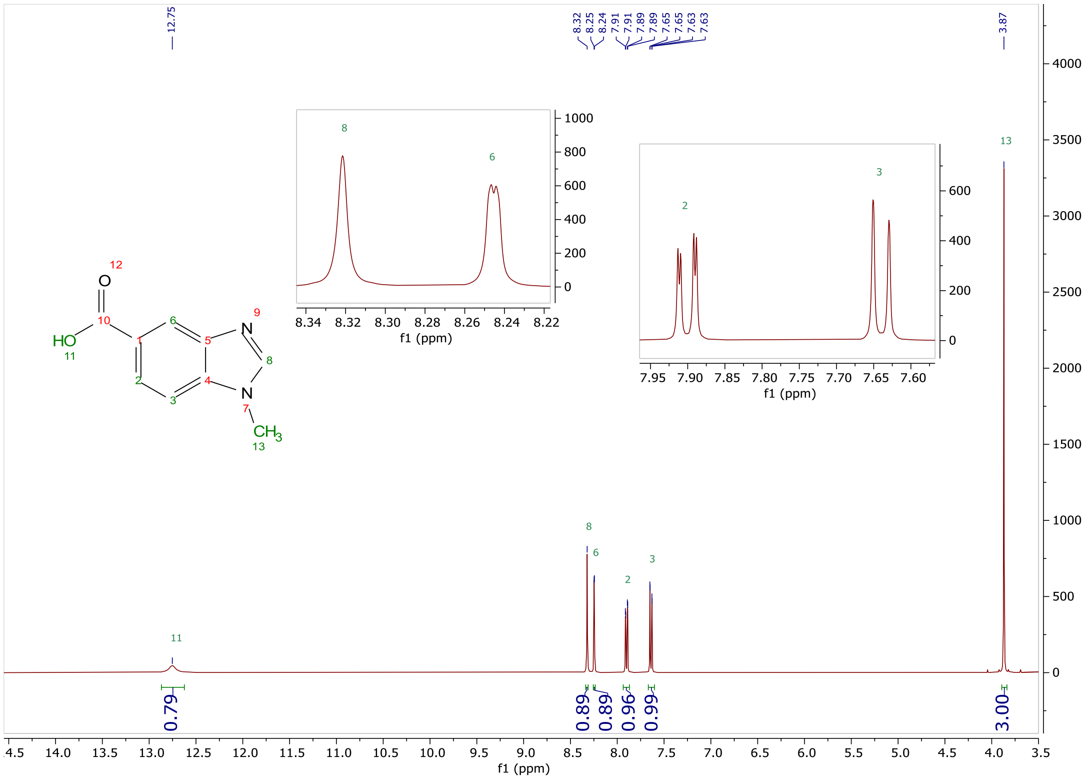

Figure 11. ^{1}H NMR (DMSO-d_6, 400 MHz): 1-Methyl-1*H*-benzimidazole-5-carboxylic acid (**1-C4**).

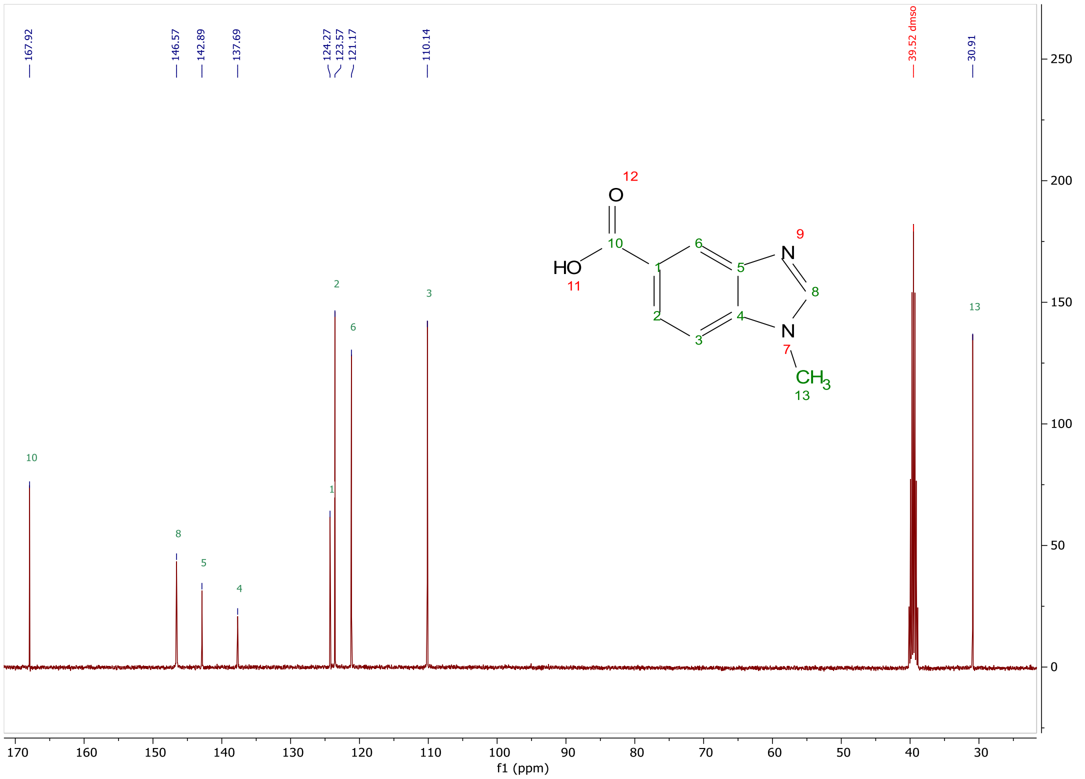

Figure 12. ^{13}C NMR (DMSO-d_6, 101 MHz): 1-Methyl-1*H*-benzimidazole-5-carboxylic acid (**1-C4**).

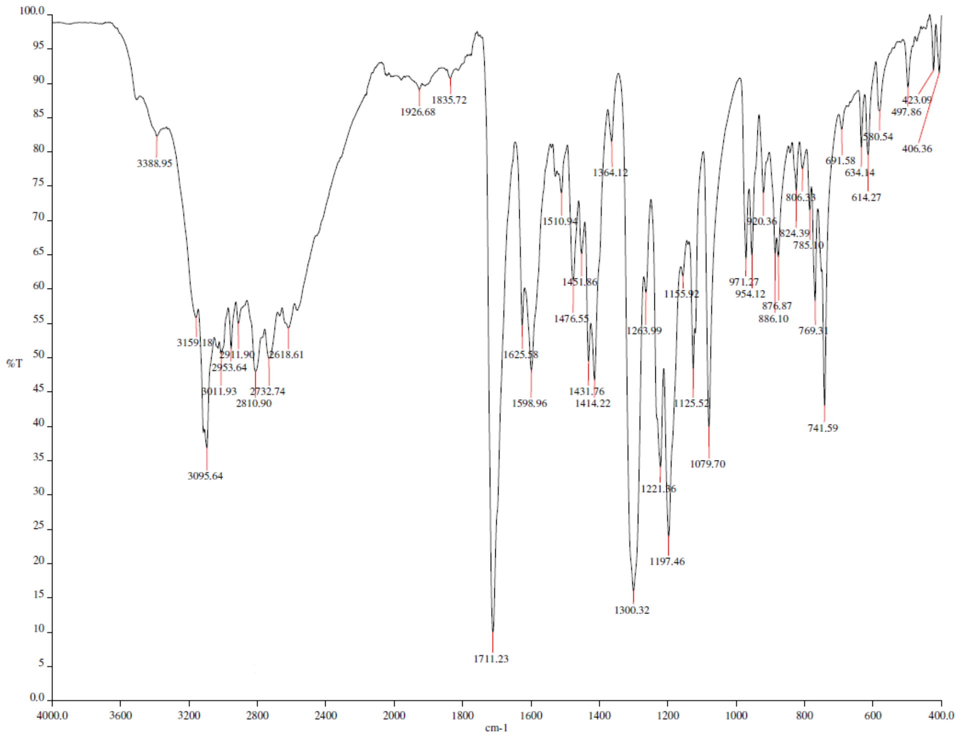

Figure 13. ATR-FTIR: Methyl 1*H*-benzimidazole-5-carboxylate (**2-C1**).

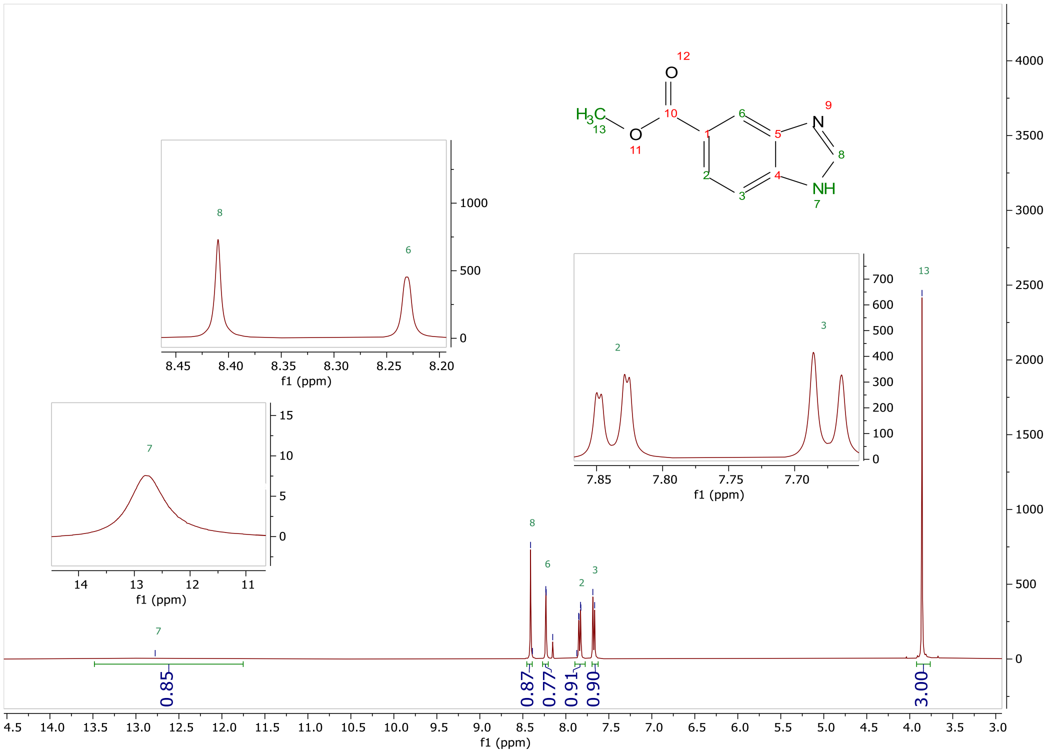

Figure 14. ^{1}H NMR (DMSO-d_6, 400 MHz): Methyl 1*H*-benzimidazole-5-carboxylate (**2-C1**).

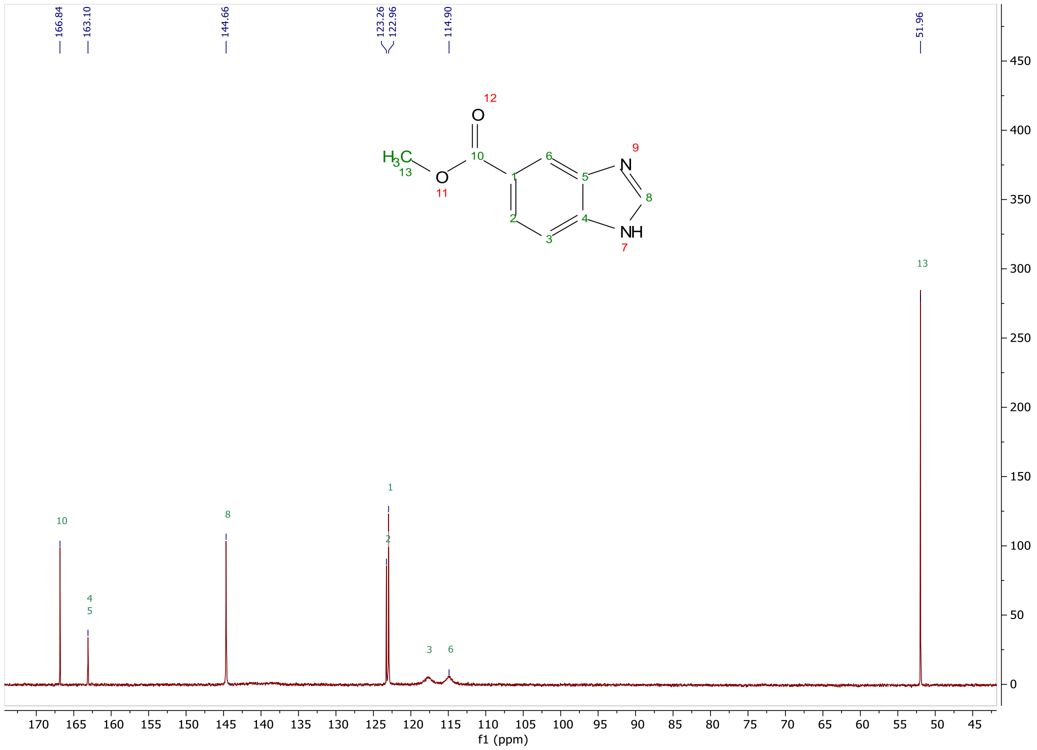

Figure 15. ^{13}C NMR (DMSO-d_6, 400 MHz): Methyl 1*H*-benzimidazole-5-carboxylate (**2-C1**).

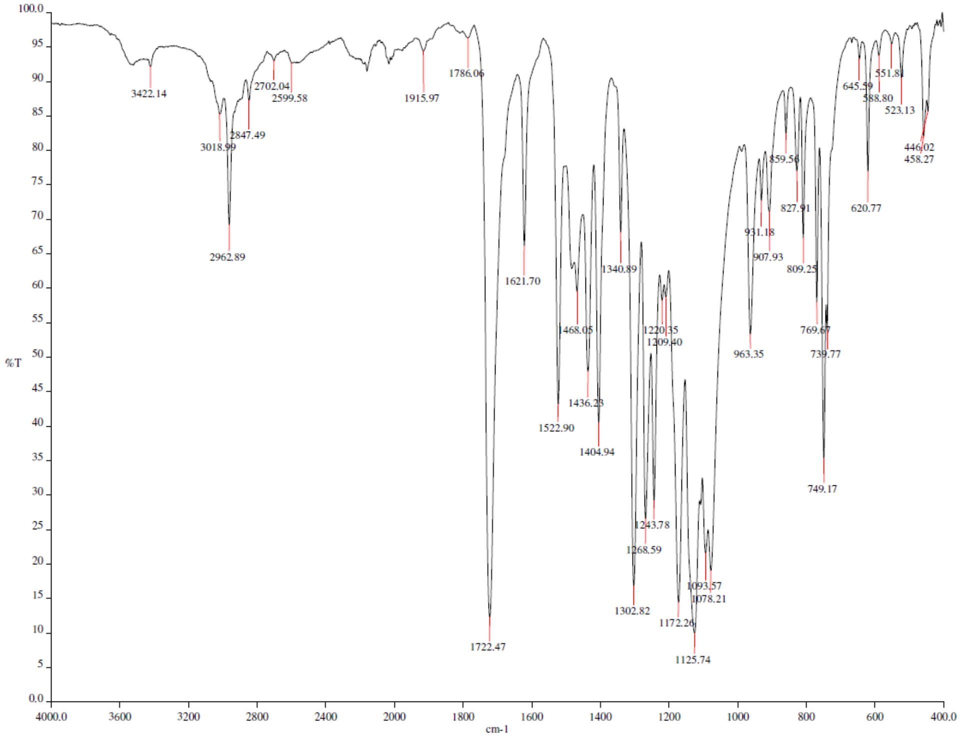

Figure 16. ATR-FTIR: Methyl 1-methyl-2-trifluoromethyl-1*H*-benzimidazole-5-carboxylate (**2-C2**).

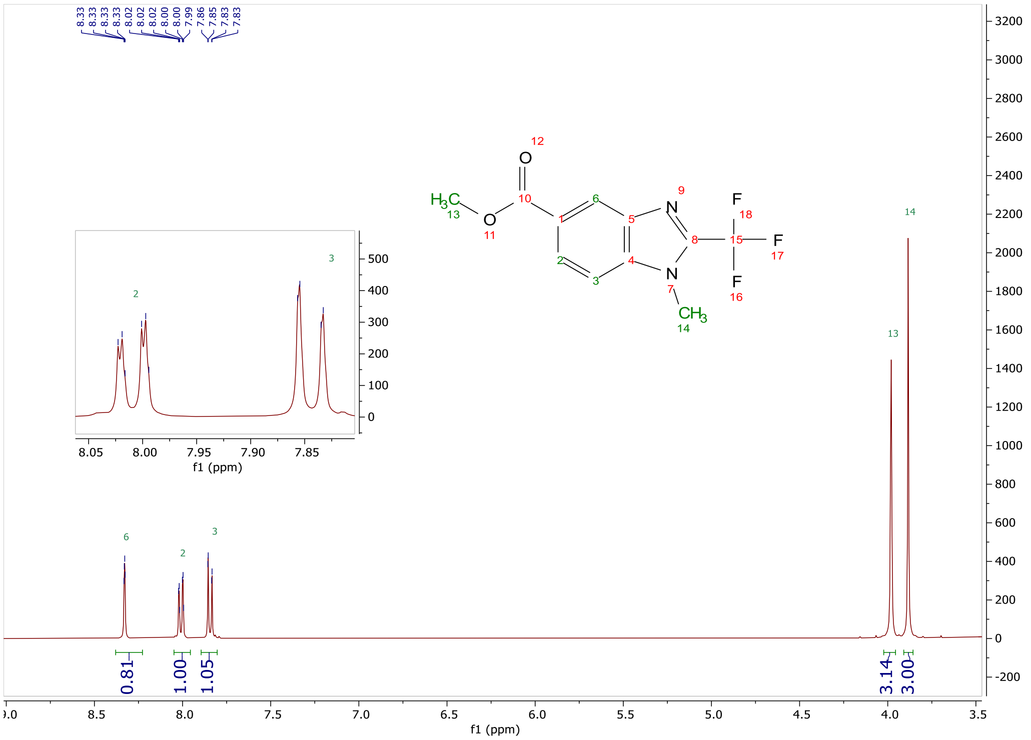

Figure 17. ^{1}H NMR (DMSO-d_6, 600 MHz): Methyl 1-methyl-2-trifluoromethyl-1*H*-benzimidazole-5-carboxylate (**2-C2**).

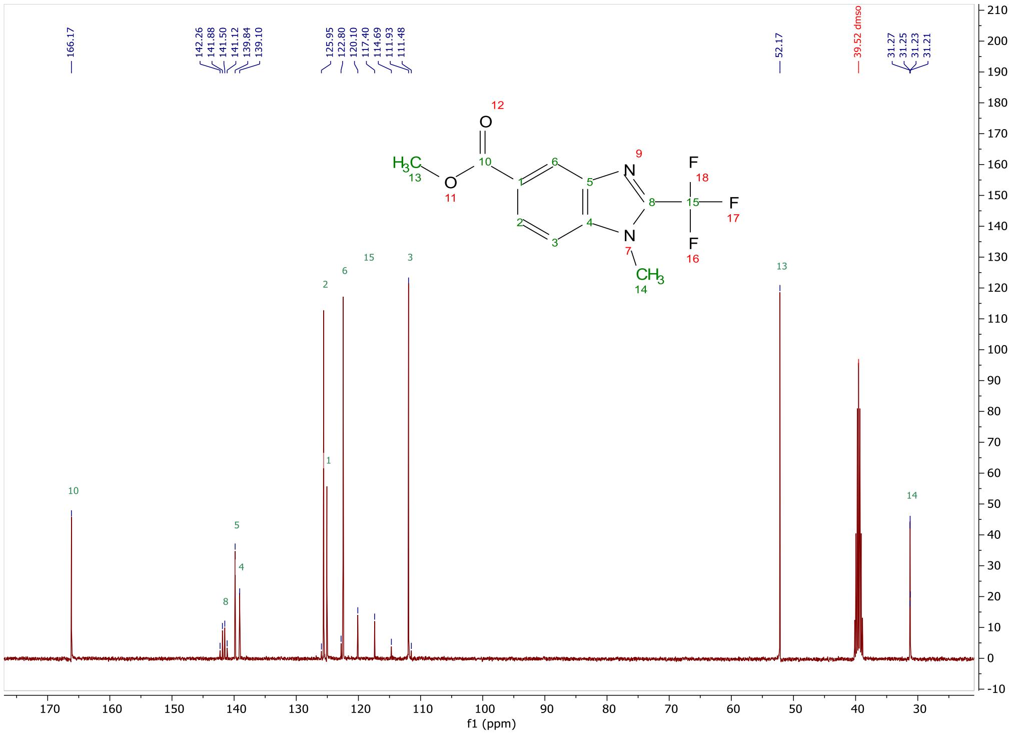

Figure 18. ^{13}C NMR (DMSO-d_6, 151 MHz): Methyl 1-methyl-2-trifluoromethyl-1*H*-benzimidazole-5-carboxylate (**2-C2**).

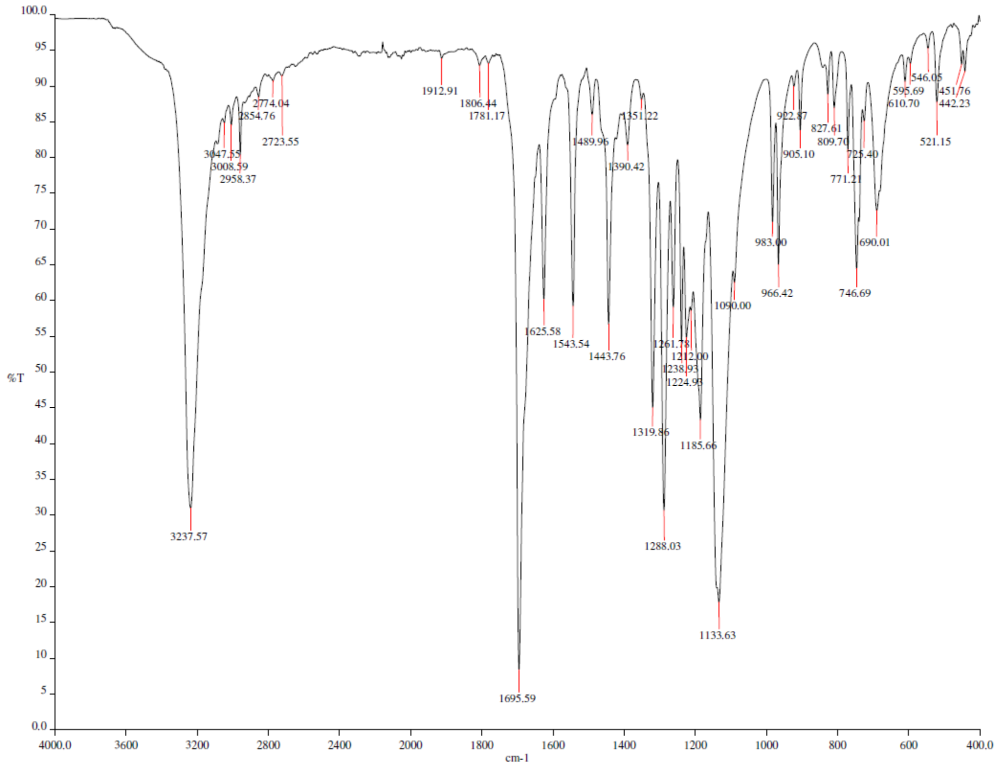

Figure 19. ATR-FTIR: Methyl 2-trifluoromethyl-1*H*-benzimidazole-5-carboxylate (**2-C3**).

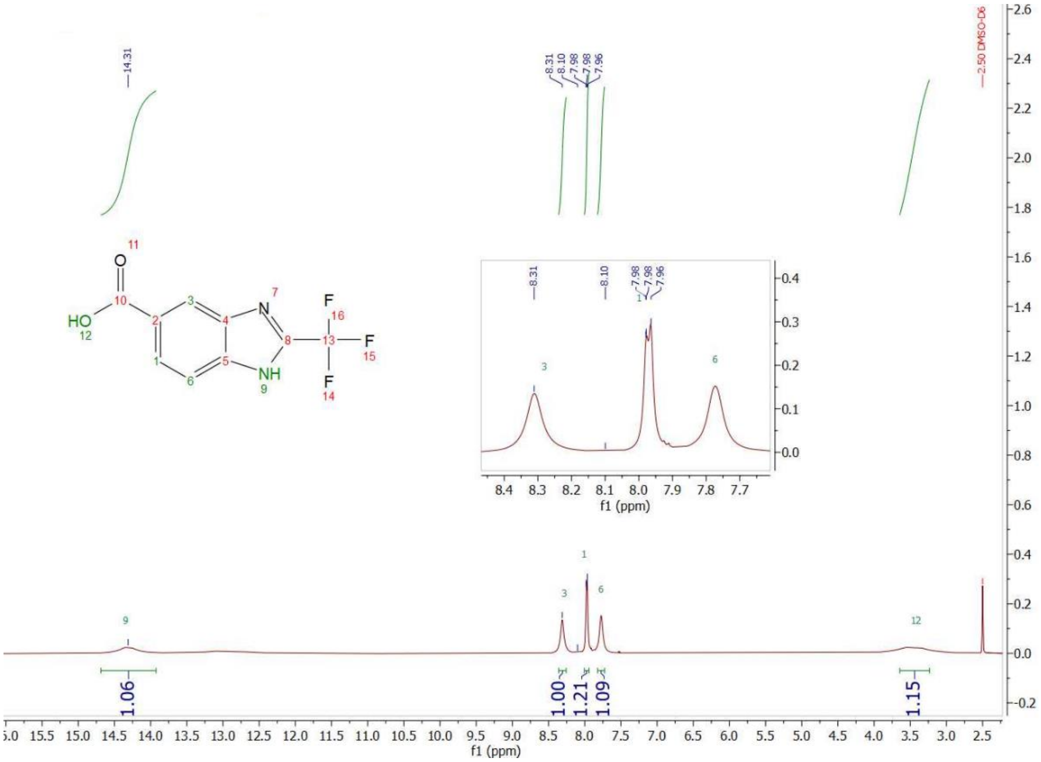

Figure 20. ^{1}H NMR (DMSO-d_6, 600 MHz): Methyl 2-trifluoromethyl-1*H*-benzimidazole-5-carboxylate (**2-C3**).

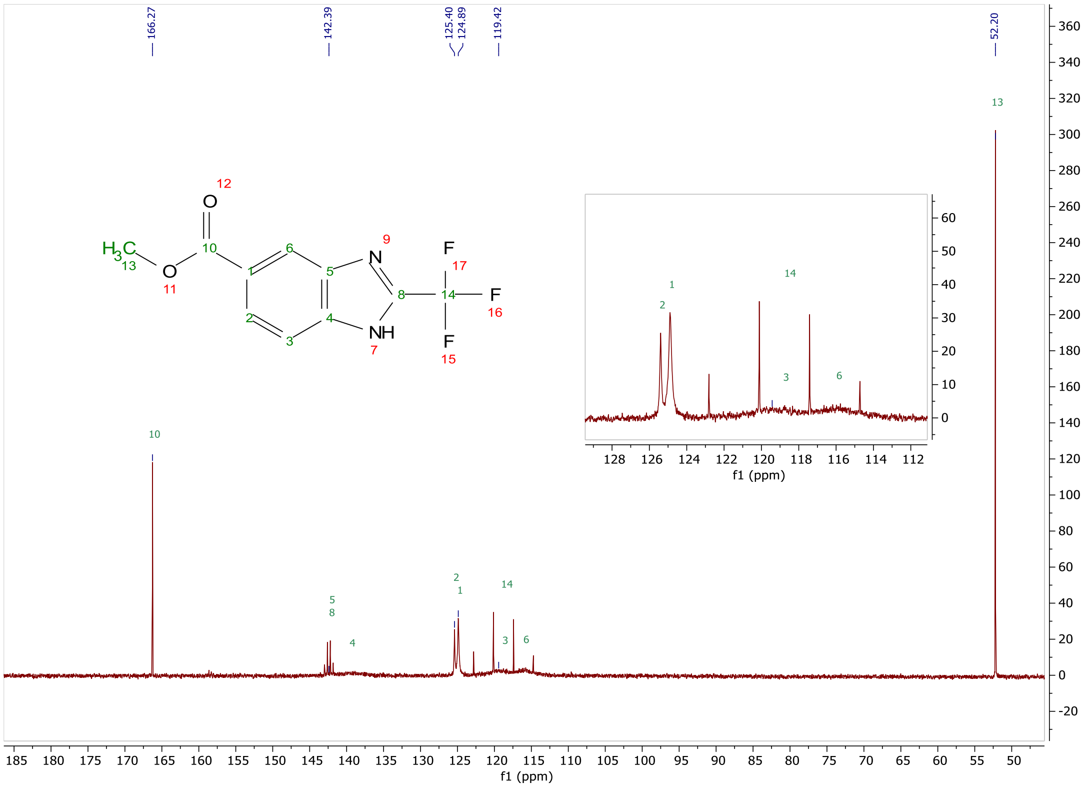

Figure 21. ^{13}C NMR (DMSO-d_6, 101 MHz): Methyl 2-trifluoromethyl-1*H*-benzimidazole-5-carboxylate (**2-C3**).

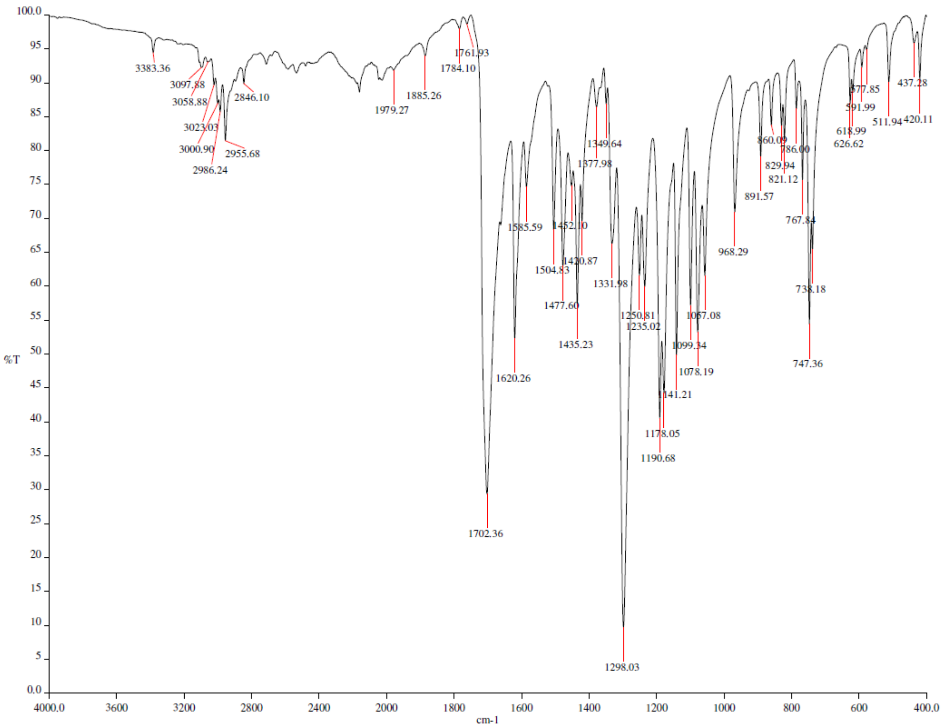

Figure 22. ATR-FTIR: Methyl 1-methyl-1*H*-benzimidazole-5-carboxylate (**2-C4**).

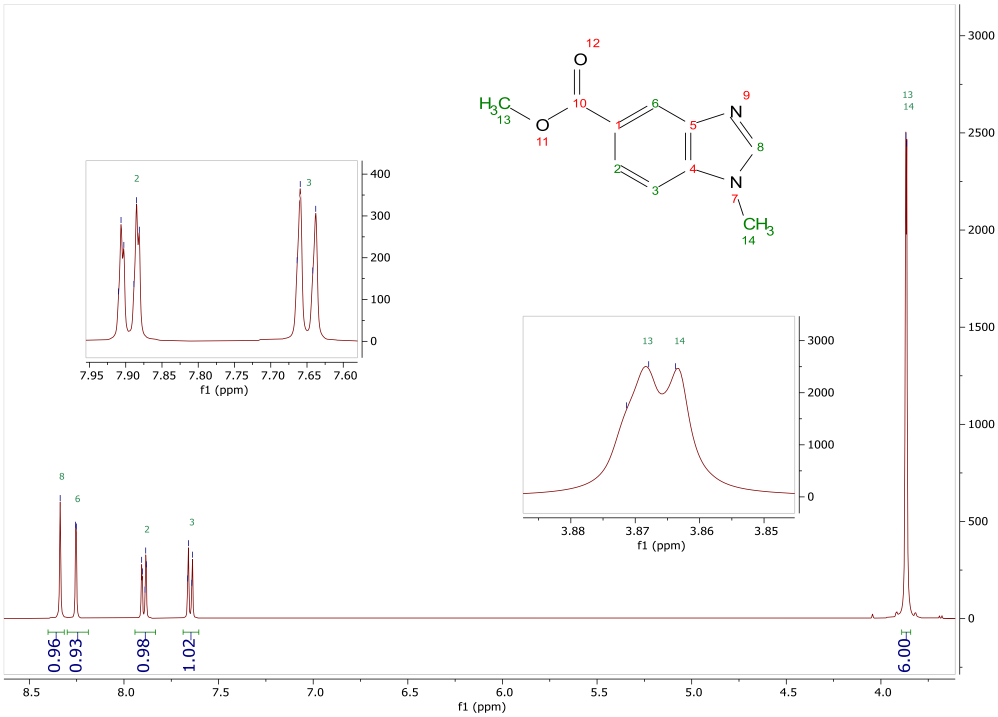

Figure 23. ^{1}H NMR (DMSO-d_6, 400 MHz): Methyl 1-methyl-1*H*-benzimidazole-5-carboxylate (**2-C4**).

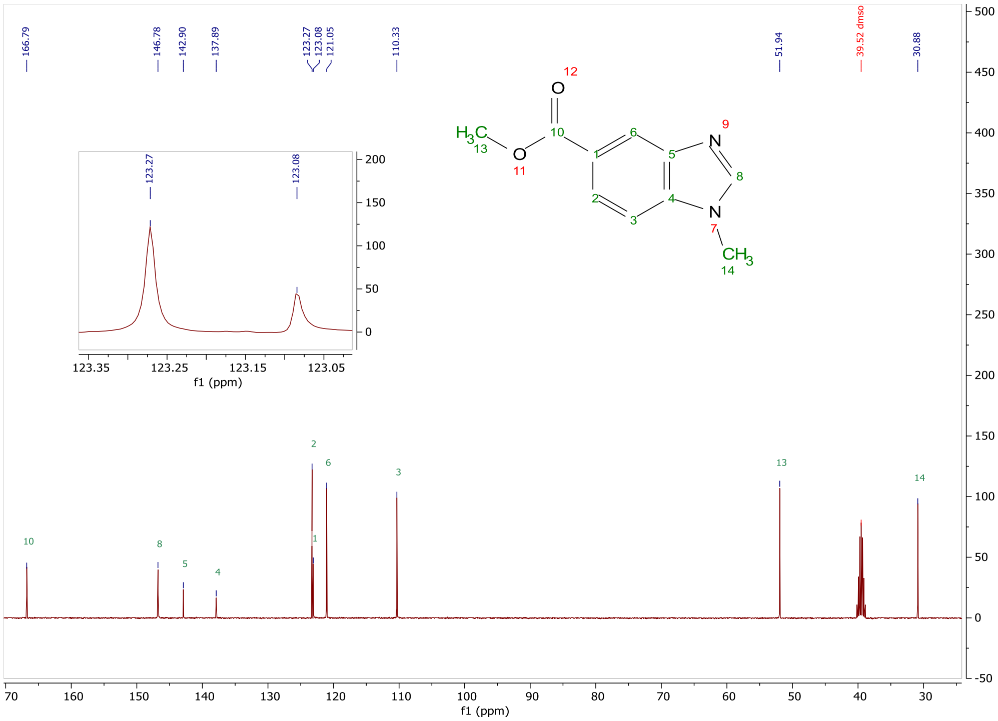

Figure 24. ^{13}C NMR (DMSO-d_6, 101 MHz): Methyl 1-methyl-1*H*-benzimidazole-5-carboxylate (**2-C4**).

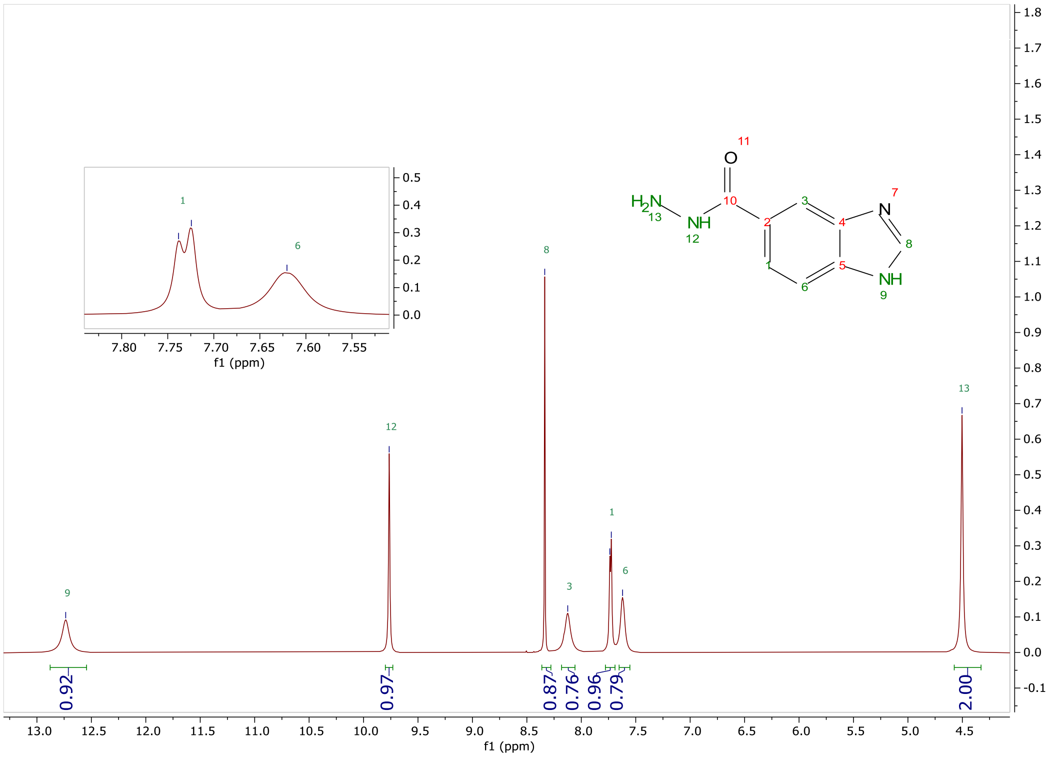

Figure 25. ^{1}H NMR (DMSO-d_6, 600 MHz): 1*H*-benzimidazole-5-carbohydrazide (**3-C1**).

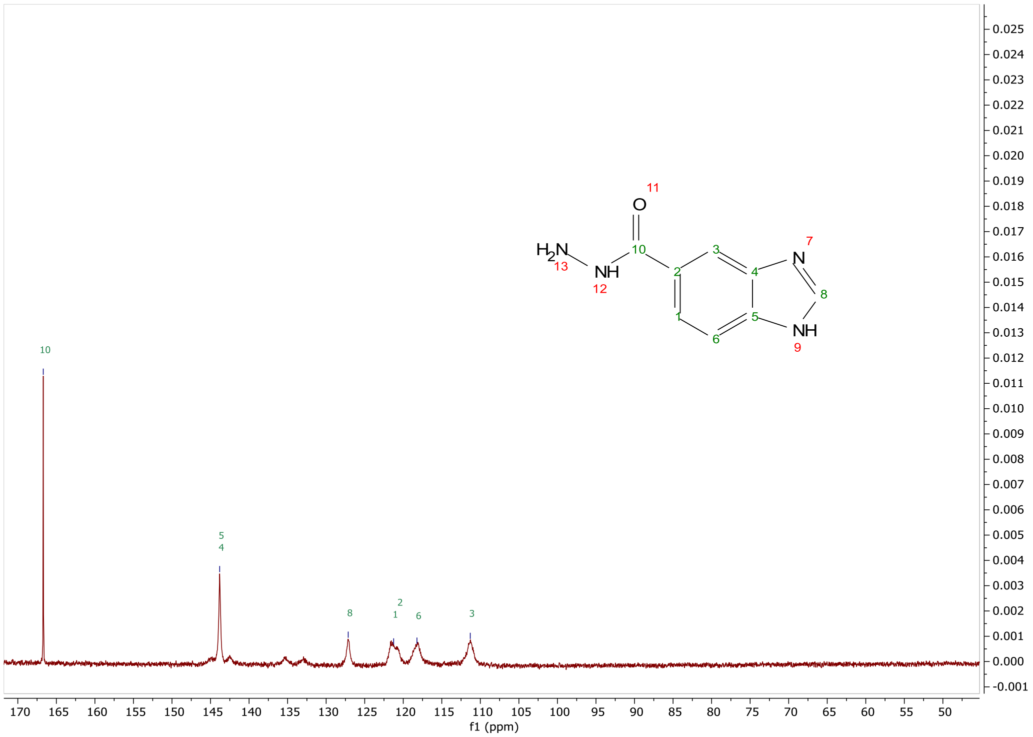

Figure 26. ^{13}C NMR (DMSO-d_6, 151 MHz): 1*H*-benzimidazole-5-carbohydrazide (**3-C1**).

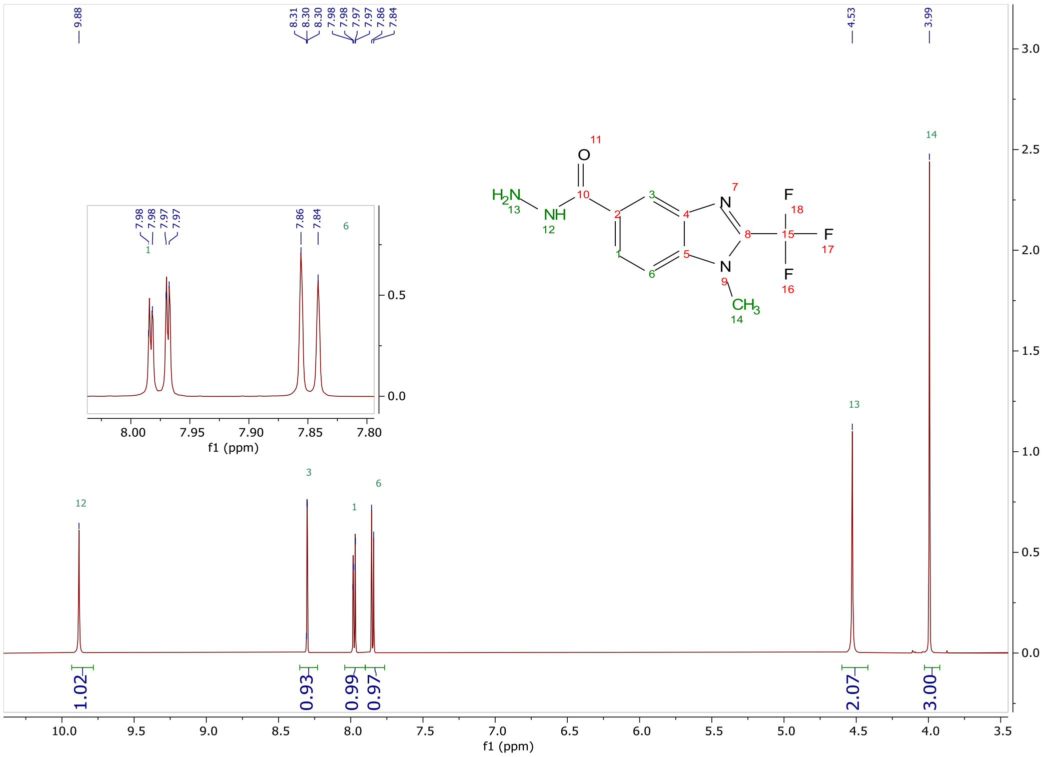

Figure 27. ^{1}H NMR (DMSO-d_6, 600 MHz): 1-Methyl-2-(trifluoromethyl)-1*H*-benzimidazole-5-carbohydrazide (**3-C2**).

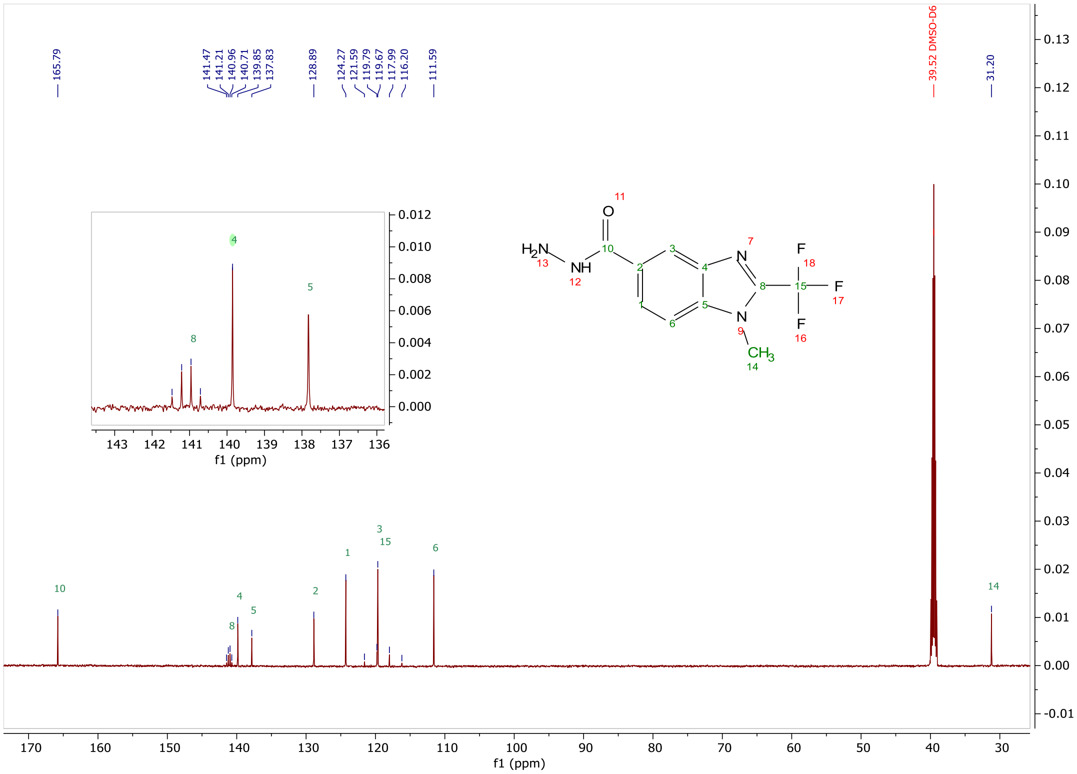

Figure 28. ^{13}C NMR (DMSO-d_6, 151 MHz): 1-Methyl-2-(trifluoromethyl)-1*H*-benzimidazole-5-carbohydrazide (**3-C2**).

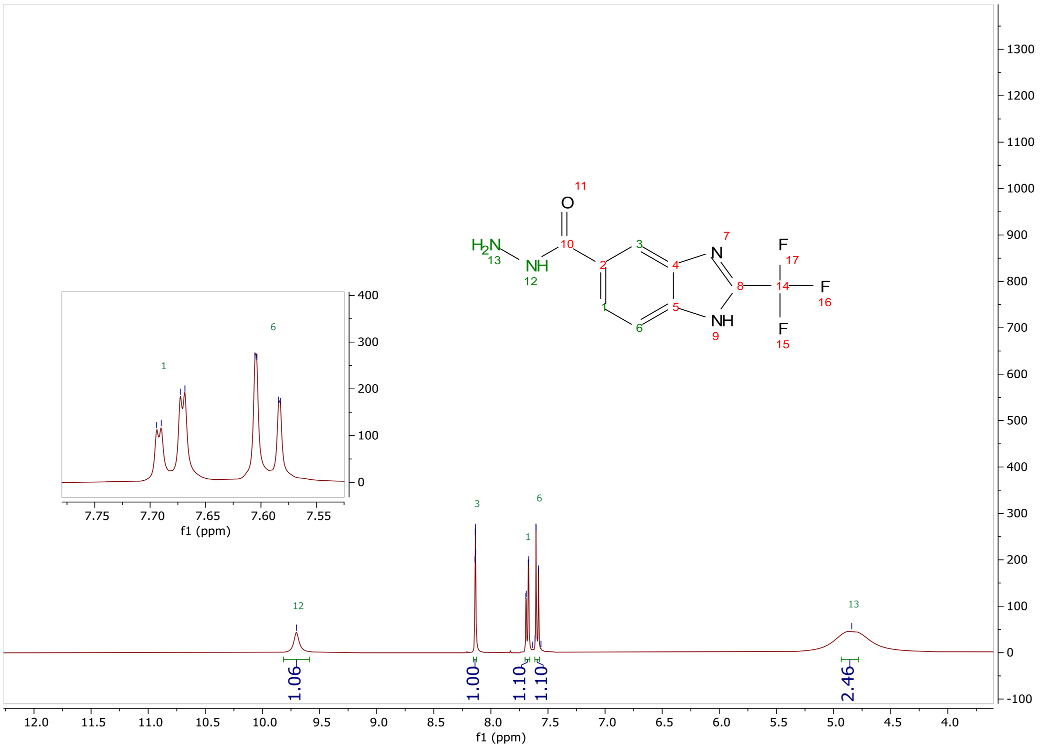

Figure 29. ^{1}H NMR (DMSO-d_6, 400 MHz): 2-(Trifluoromethyl)-1*H*-benzimidazole-5-carbohydrazide (**3-C3**).

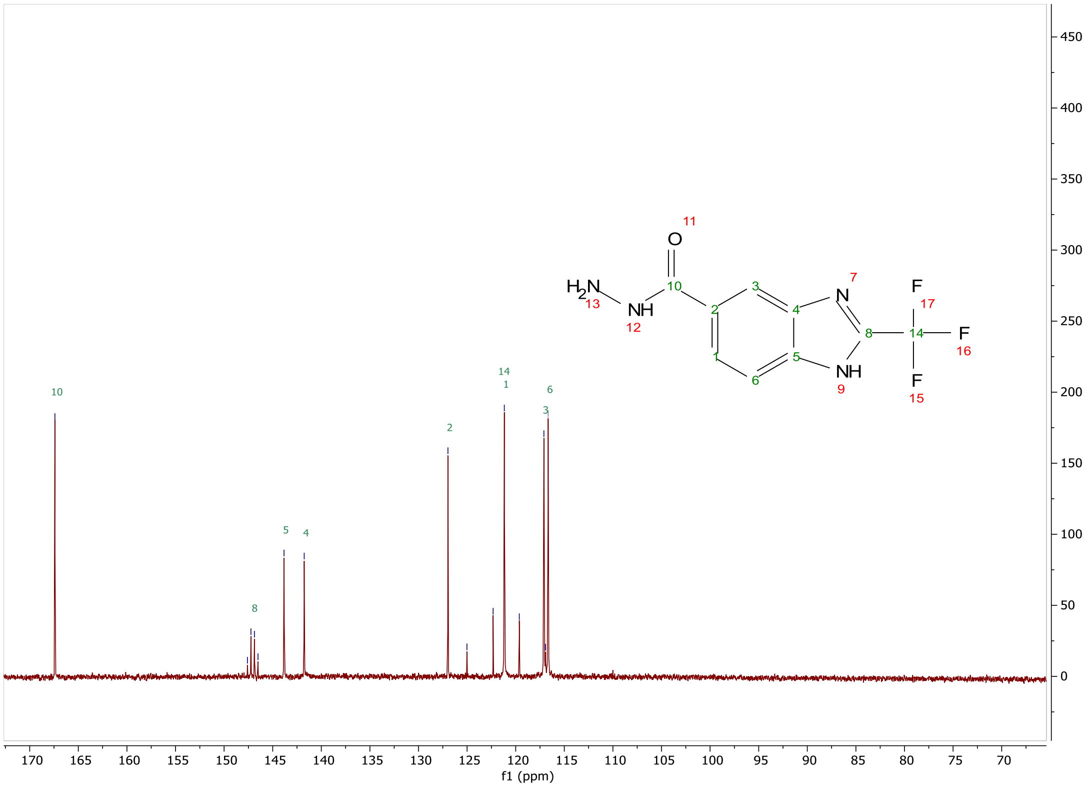

Figure 30. ^{13}C NMR (DMSO-d_6, 101 MHz): 2-(Trifluoromethyl)-1*H*-benzimidazole-5-carbohydrazide (**3-C3**).

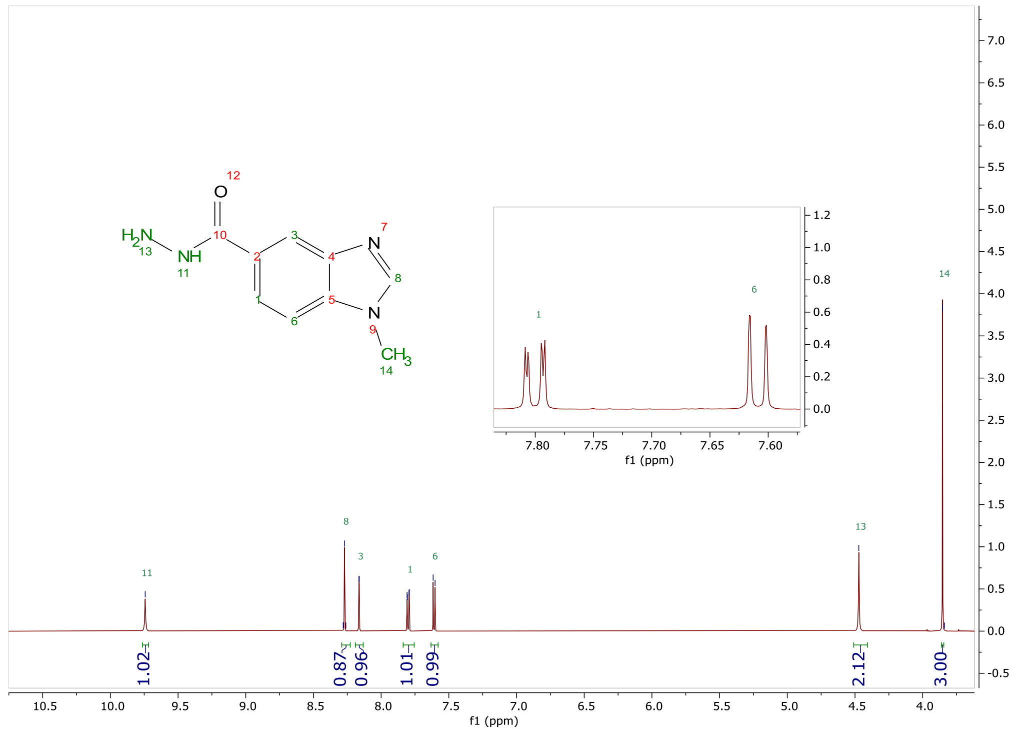

Figure 31. ^{1}H NMR (DMSO-d_6, 600 MHz): 1-Methyl-1*H*-benzimidazole-5-carbohydrazide (**3-C4**).

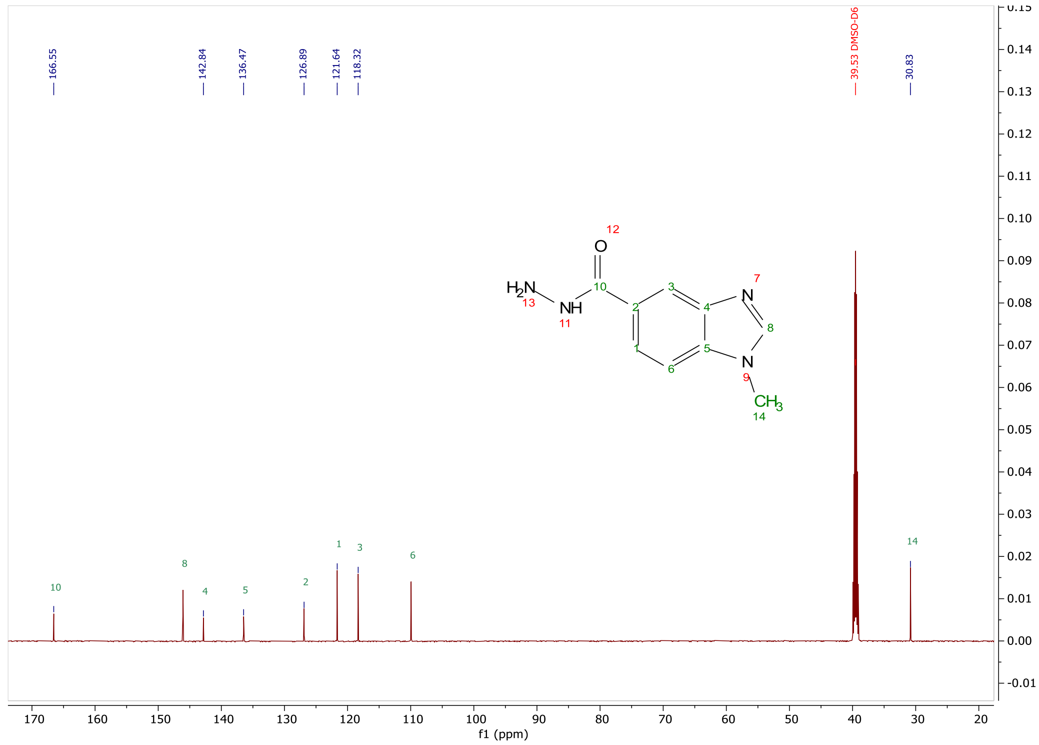

Figure 32. ^{13}C NMR (DMSO-d_6, 151 MHz): 1-Methyl-1*H*-benzimidazole-5-carbohydrazide (**3-C4**).

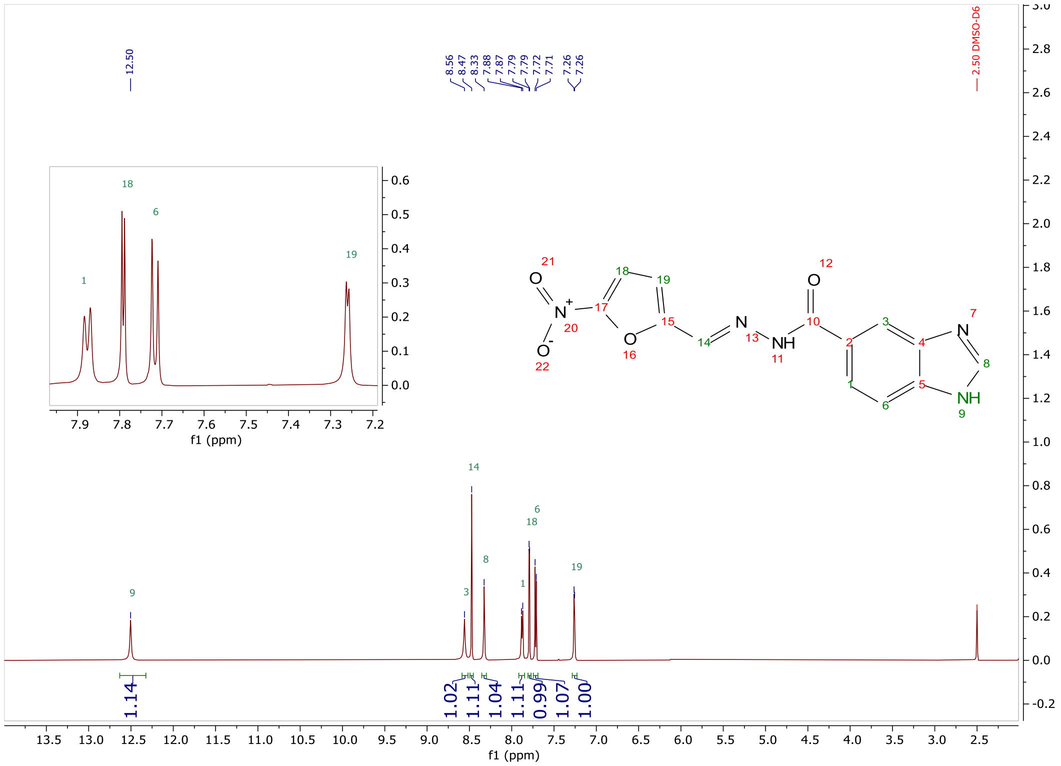

Figure 33. ^{1}H NMR (DMSO-d_6, 600 MHz): *N*'-[(5-Nitrofuran-2-yl)methylene]-1*H*-benzimidazole-5-carbohydrazide (**C1**).

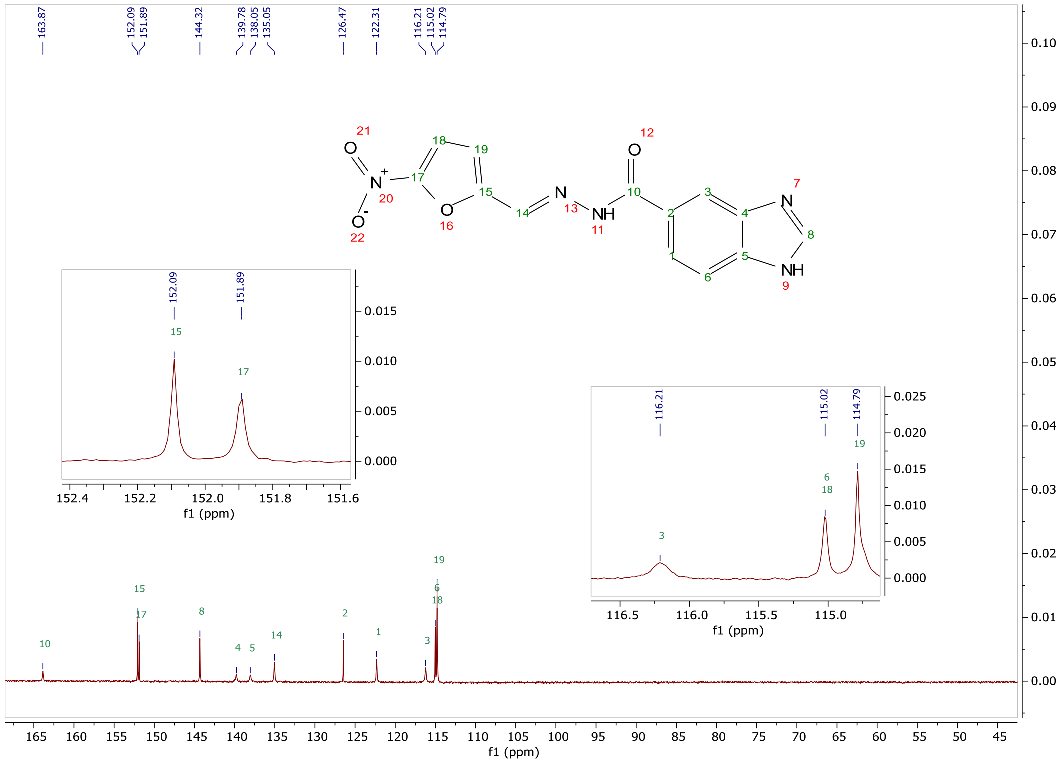

Figure 34. ^{13}C NMR (DMSO-d_6, 151 MHz): *N*'-[(5-Nitrofuran-2-yl)methylene]-1*H*-benzimidazole-5-carbohydrazide (**C1**).

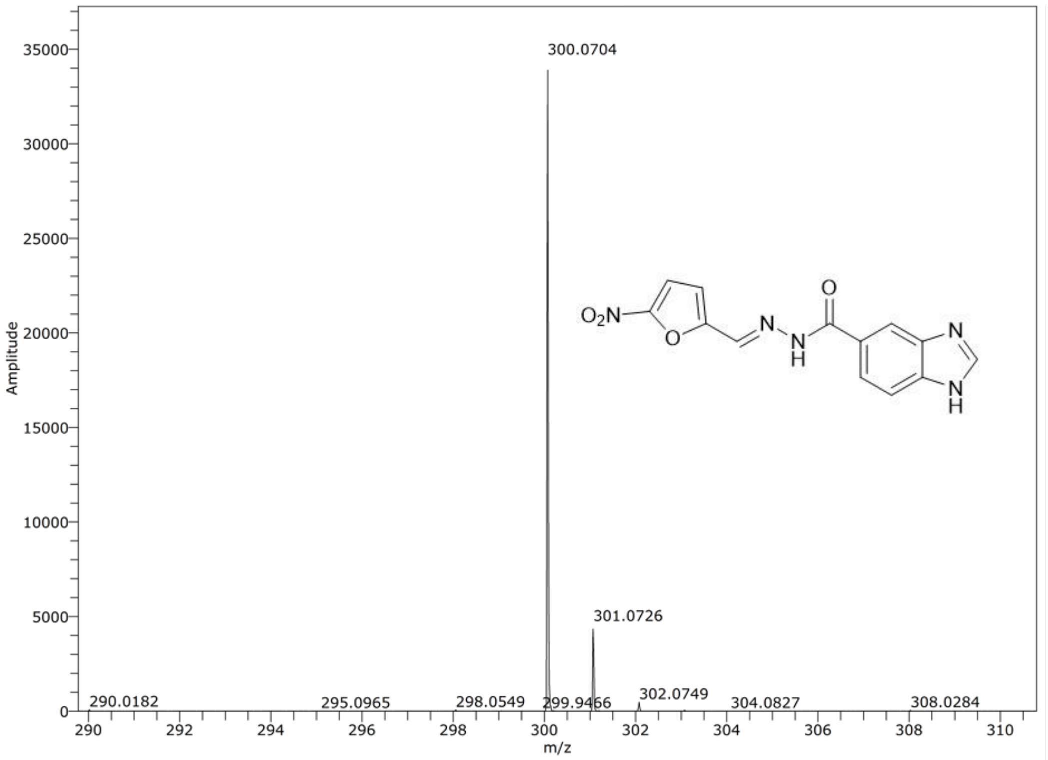

Formula	Calculated	Obtained	Obtained formula	%	ppM error	Score
$C_{13}H_9N_5O_4$	300.07273	300.0704	$C_{13}H_9N_5O_4 + H^+$	300.0704 (100), 301.0726 (18), 302.0749 (2)	-3.23	0.84

Figure 35. HRMS (APCI): *m*/*z* 300.0704 [M^+ + H] *N*'-[(5-Nitrofuran-2-yl)methylene]-1*H*-benzimidazole-5-carbohydrazide (**C1**).

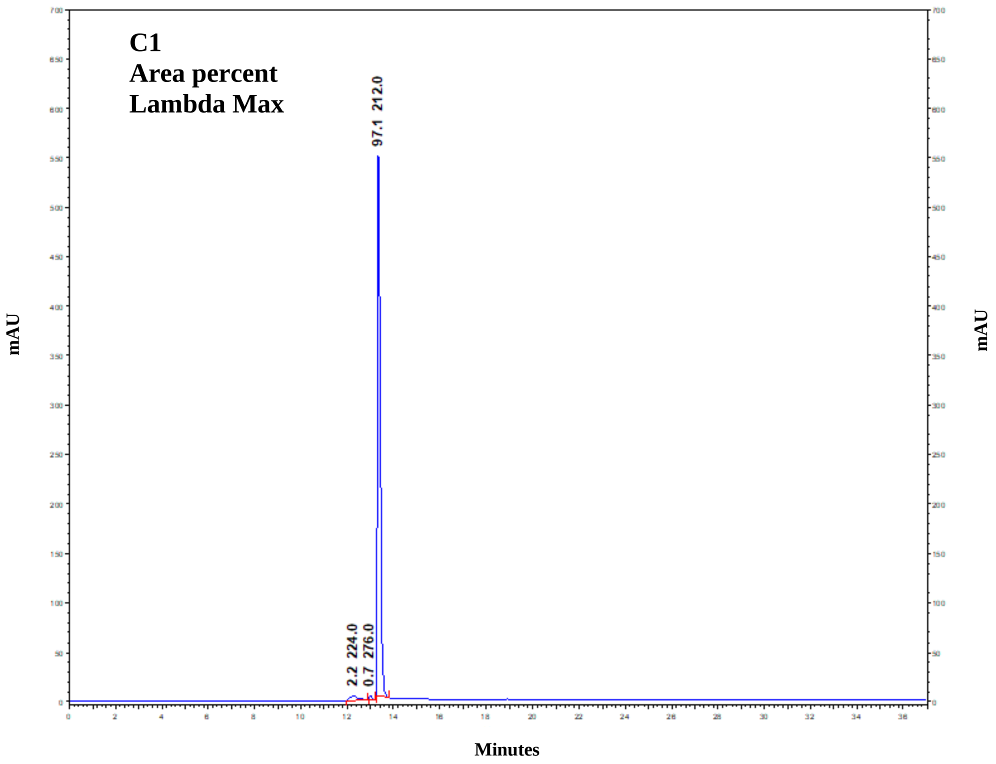

Figure 36. HPLC-DAD: 97.1% purity, with a λmax of 212.0 nm as a result of scanning from 190 to 600 nm of *N*'-[(5-Nitrofuran-2-yl)methylene]-1*H*-benzimidazole-5-carbohydrazide (**C1**).

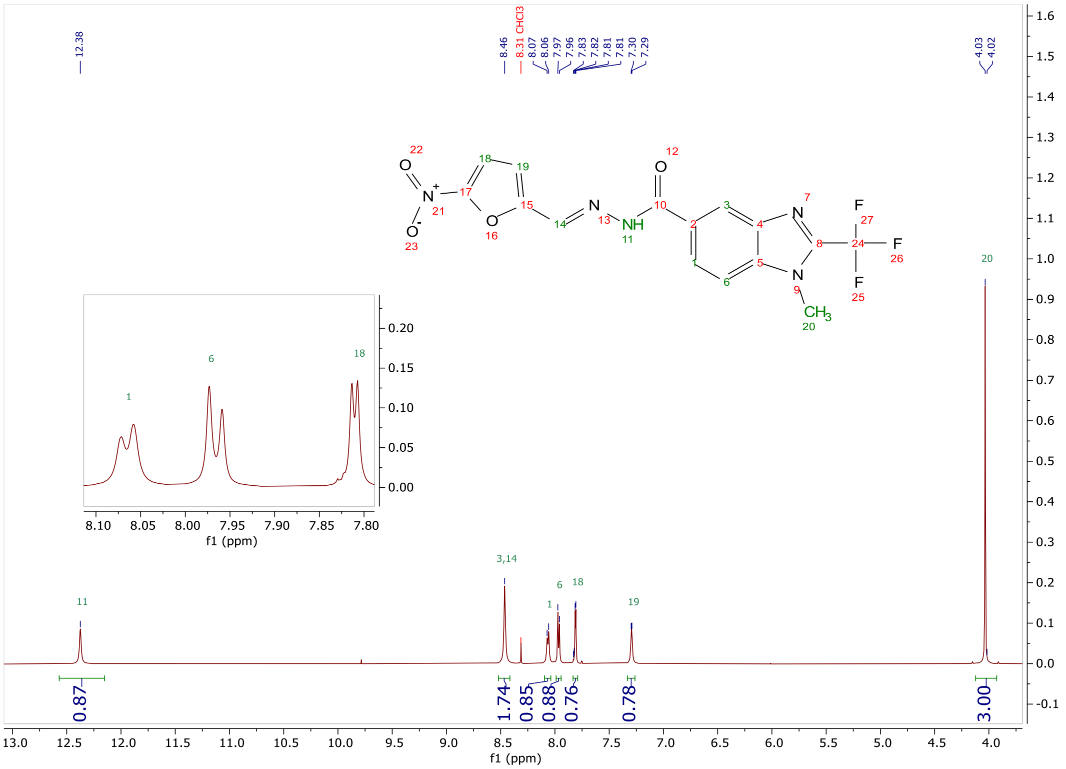

Figure 37. ^{1}H NMR (DMSO-d_6, 600 MHz): 1-Methyl-*N*'-[(5-nitrofuran-2-yl)methylene]-2-(trifluoromethyl)-1*H*-benzimidazole-5-carbohydrazide (**C2**).

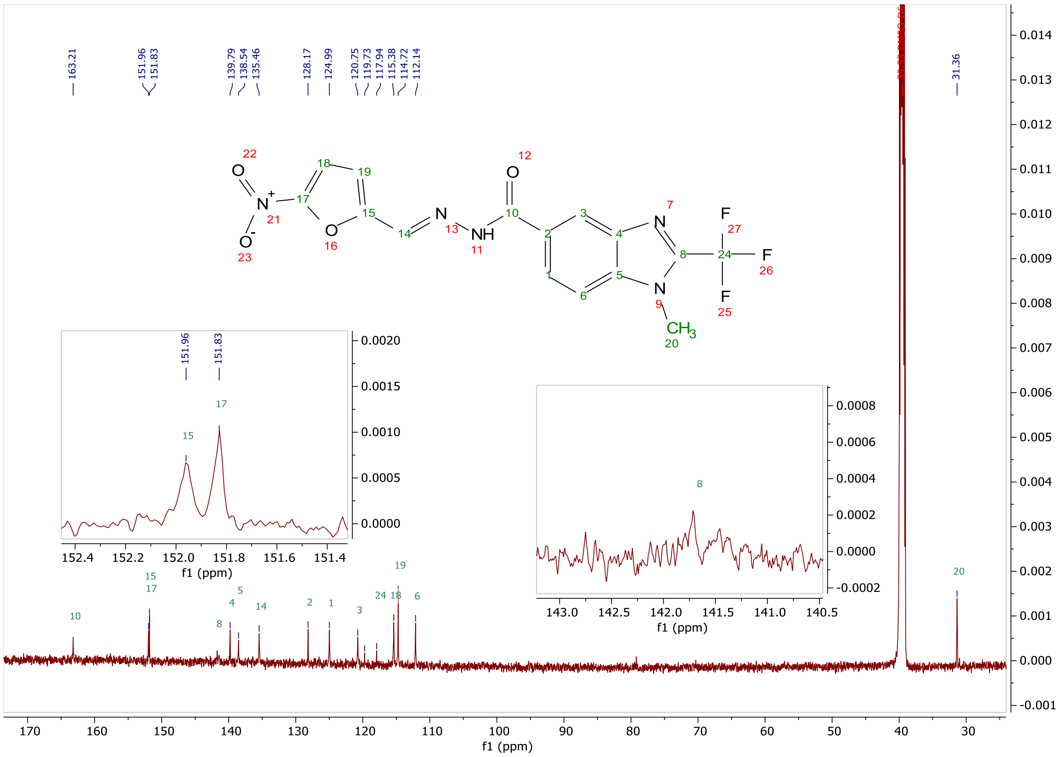

Figure 38. ^{13}C NMR (DMSO-d_6, 151 MHz): 1-Methyl-*N*'-[(5-nitrofuran-2-yl)methylene]-2-(trifluoromethyl)-1*H*-benzimidazole-5-carbohydrazide (**C2**).

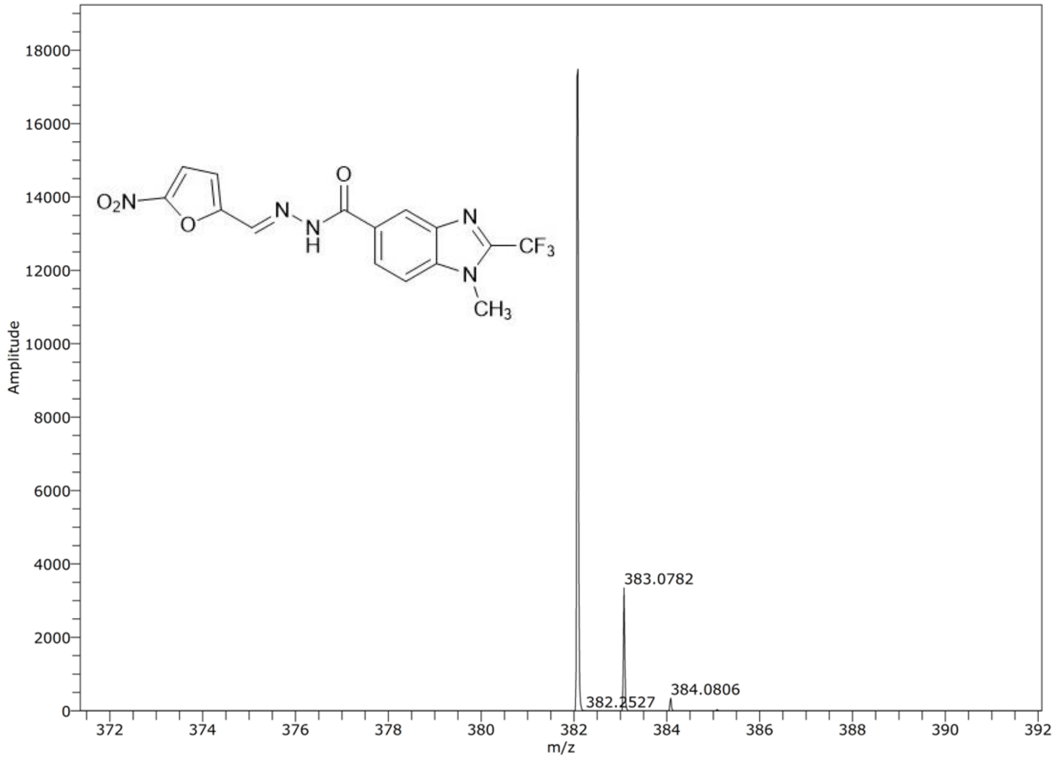

Formula	Calculated	Obtained	Obtained formula	%	ppM error	Score
$C_{15}H_{10}F_3N_5O_4$	382.07577	382.0761	$C_{15}H_{10}F_3N_5O_4 + H^+$	382.0761 (100), 383.0782 (18), 384.0806 (2)	-0.87	0.849

Figure 39. HRMS (APCI): *m*/*z* 382.0761 [M⁺ + H] 1-Methyl-*N*'-[(5-nitrofuran-2-yl)methylene]-2-(trifluoromethyl)-1*H*-benzimidazole-5-carbohydrazide (**C2**).

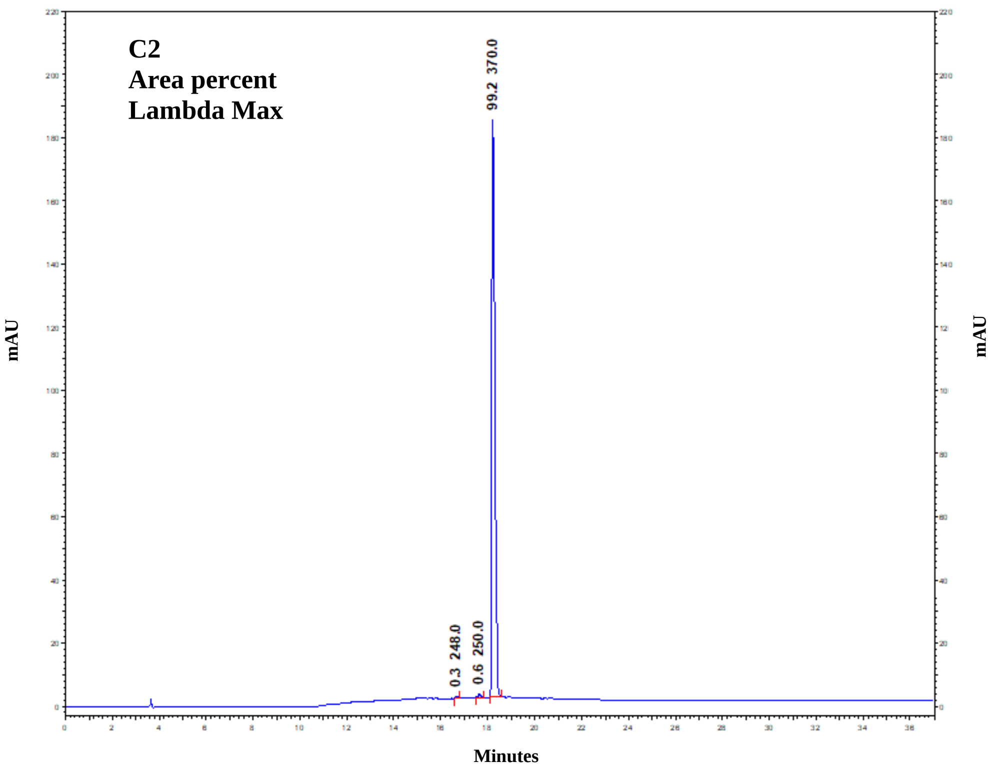

Figure 40. HPLC-DAD: 99.2% purity, with a *λ*max of 370.0 nm as a result of scanning from 190 to 600 nm of 1-Methyl-*N*'-[(5-nitrofuran-2-yl)methylene]-2-(trifluoromethyl)-1*H*-benzimidazole-5-carbohydrazide (**C2**).

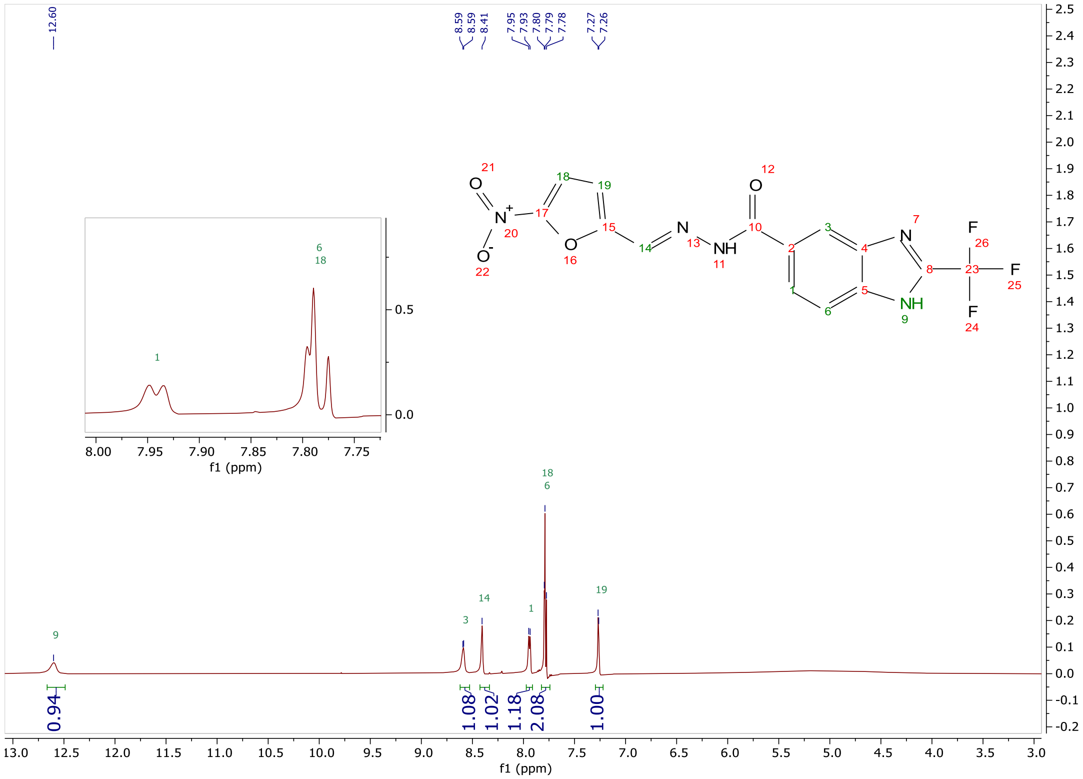

Figure 41. ^{1}H NMR (DMSO-d_6, 600 MHz): *N*'-[(5-Nitrofuran-2-yl)methylene]-2-(trifluoromethyl)-1*H*-benzimidazole-5-carbohydrazide (**C3**).

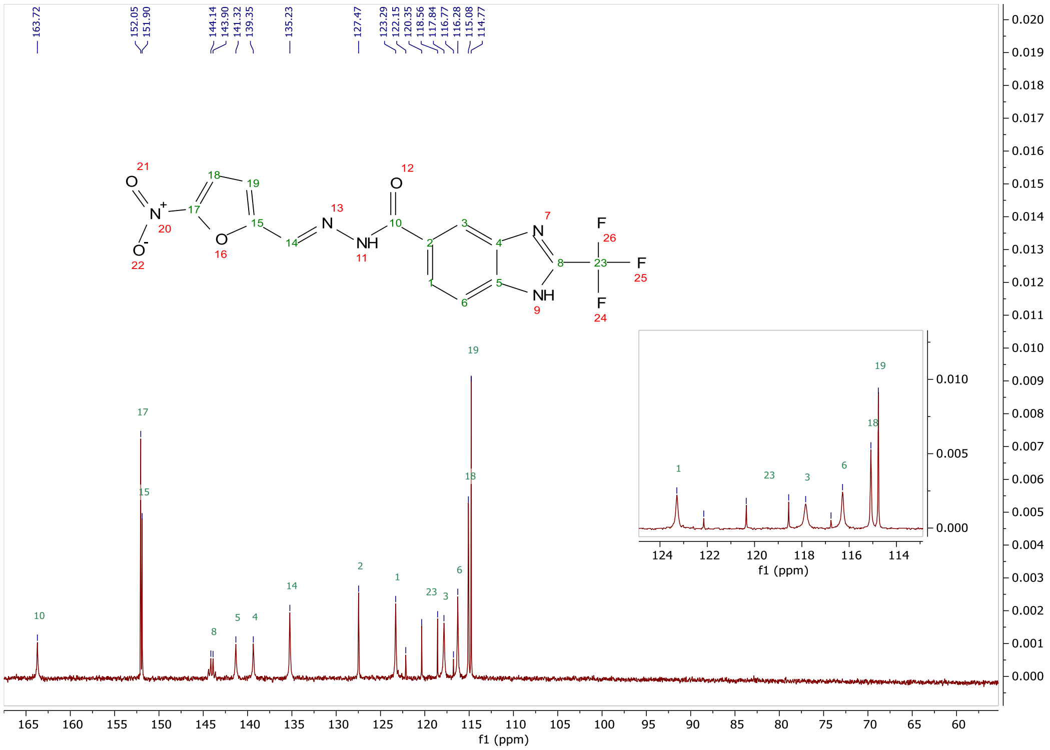

Figure 42. ^{13}C NMR (DMSO-d_6, 151 MHz): *N*'-[(5-Nitrofuran-2-yl)methylene]-2-(trifluoromethyl)-1*H*-benzimidazole-5-carbohydrazide (**C3**).

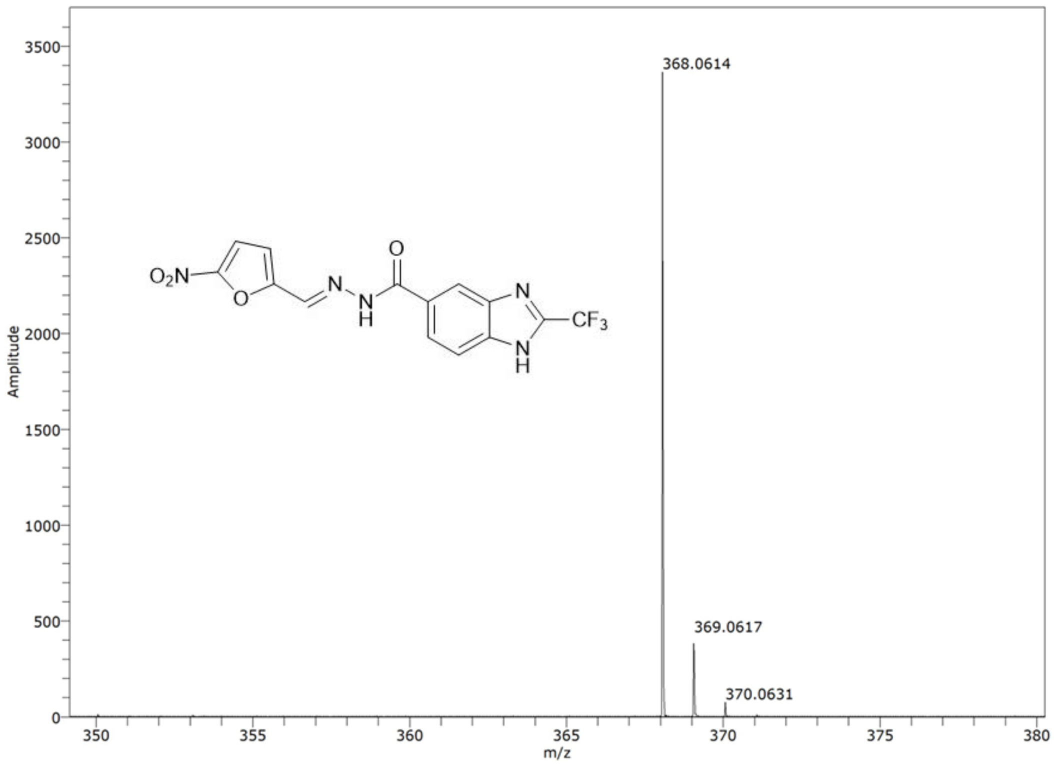

Formula	Calculated	Obtained	Obtained formula	%	ppM error	Score
$C_{14}H_8F_3N_5O_4$	368.06012	368.0614	$C_{14}H_8F_3N_5O_4 + H^+$	368.0614 (100), 369.0617 (18), 370.0631 (2)	-3.08	0.804

Figure 43. HRMS (APCI): *m/z* 368.0614 [M^+ + H] *N'*-[(5-Nitrofuran-2-yl)methylene]-2-(trifluoromethyl)-1*H*-benzimidazole-5-carbohydrazide (**C3**).

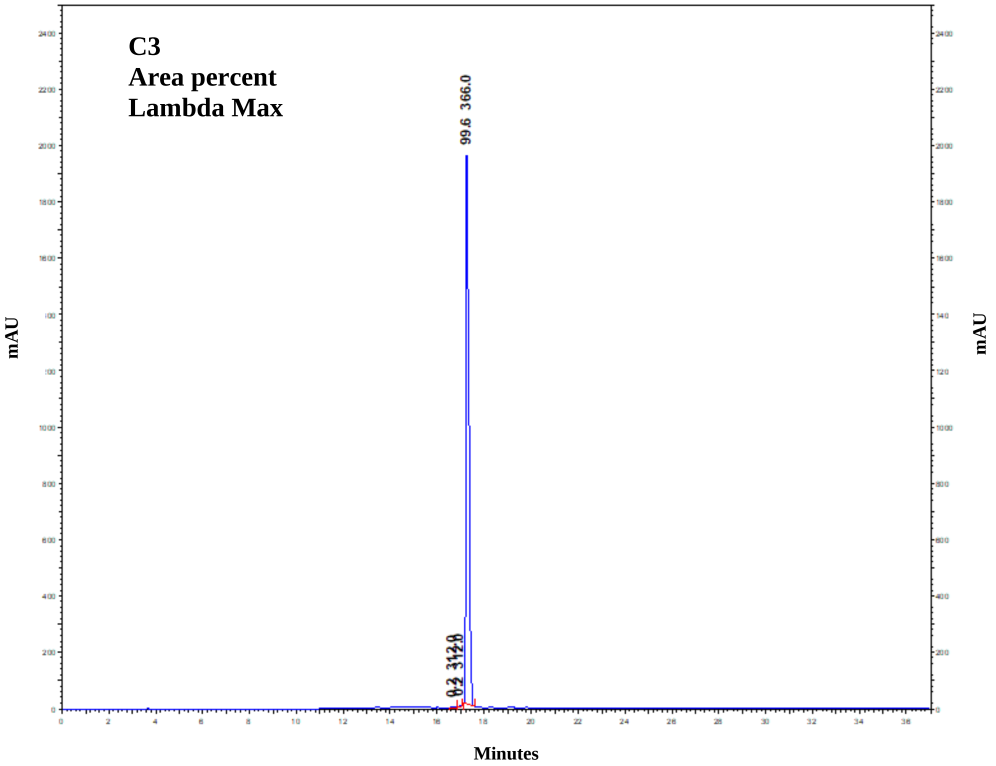

Figure 44. HPLC-DAD: 99.6% purity, with a λmax of 366.0 nm as a result of scanning from 190 to 600 nm of *N*'-[(5-Nitrofuran-2-yl)methylene]-2-(trifluoromethyl)-1*H*-benzimidazole-5-carbohydrazide (**C3**).

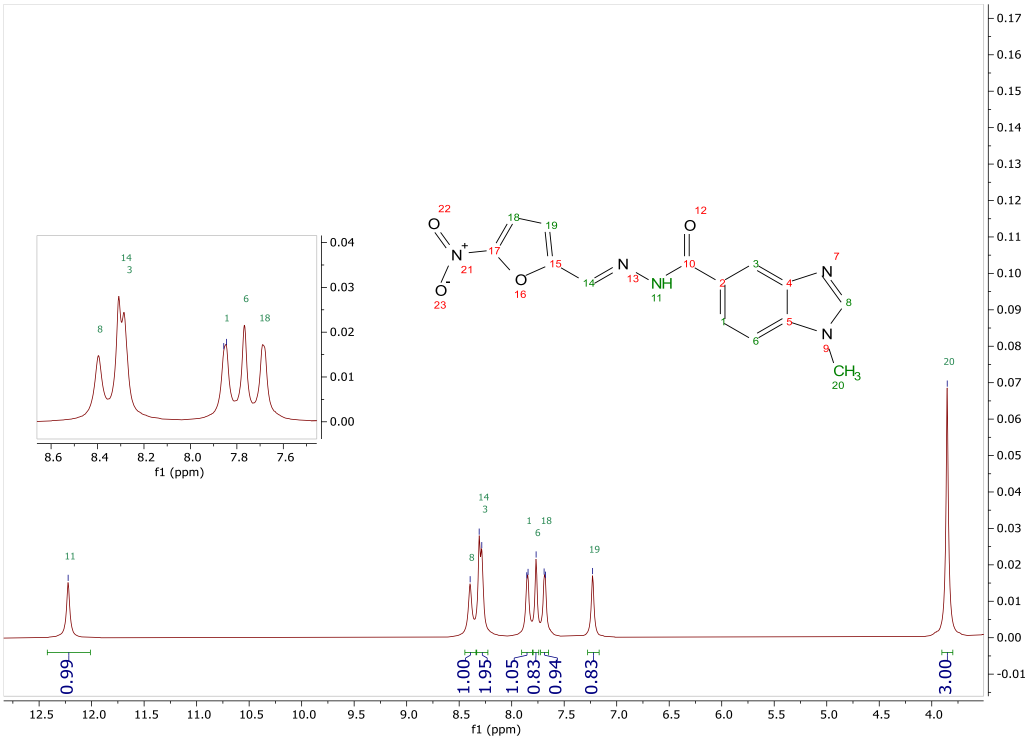

Figure 45. ^{1}H NMR (DMSO-d_6, 600 MHz): 1-Methyl-*N*'-[(5-nitrofuran-2-yl)methylene]-1*H*-benzimidazole-5-carbohydrazide (**C4**).

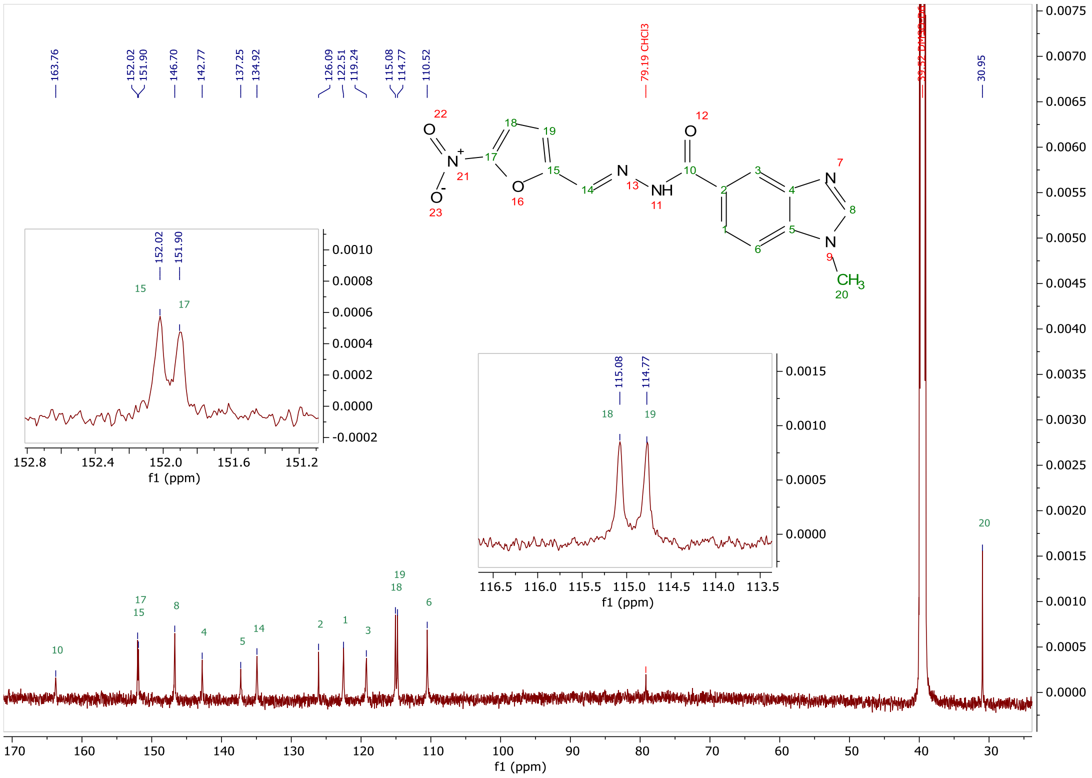

Figure 46. ^{13}C NMR (DMSO-d_6, 151 MHz): 1-Methyl-*N*'-[(5-nitrofuran-2-yl)methylene]-1*H*-benzimidazole-5-carbohydrazide (**C4**).

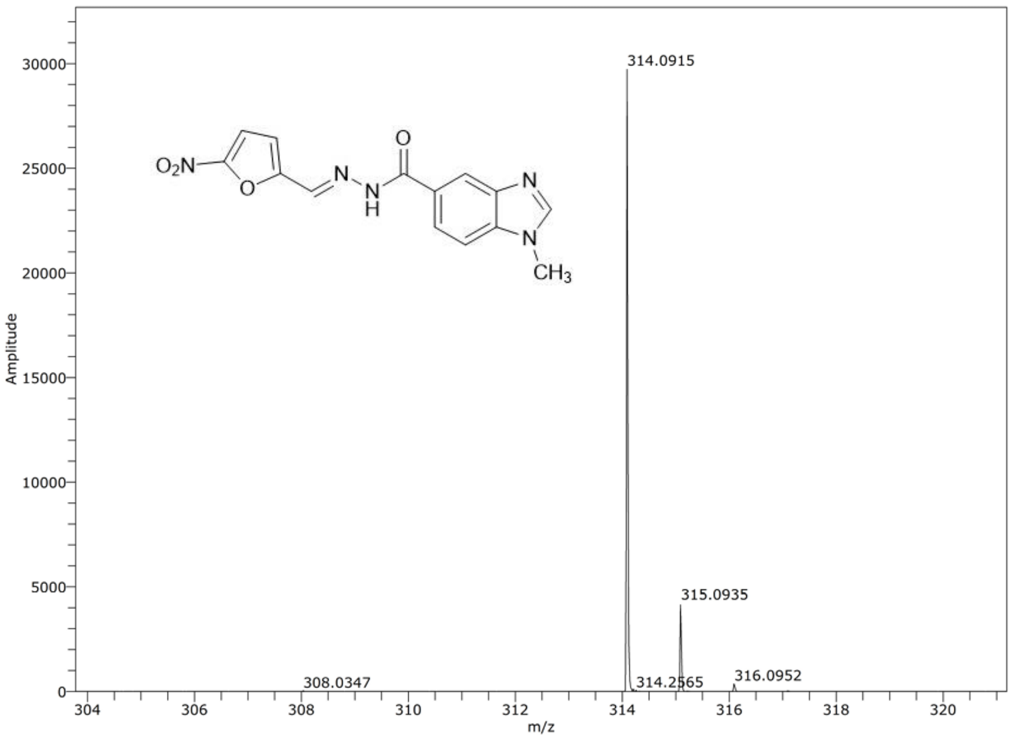

Formula	Calculated	Obtained	Obtained formula	%	ppM error	Score
$C_{14}H_{11}N_5O_4$	314.0915	314.08838	$C_{14}H_{11}N_5O_4$ + H^+	314.0915 (100), 315.0935 (18), 316.0952 (2)	-10	0.753

Figure 47. HRMS (APCI): *m*/*z* 314.0915 [M^+ + H] 1-Methyl-*N*'-[(5-nitrofuran-2-yl)methylene]-1*H*-benzimidazole-5-carbohydrazide (**C4**).

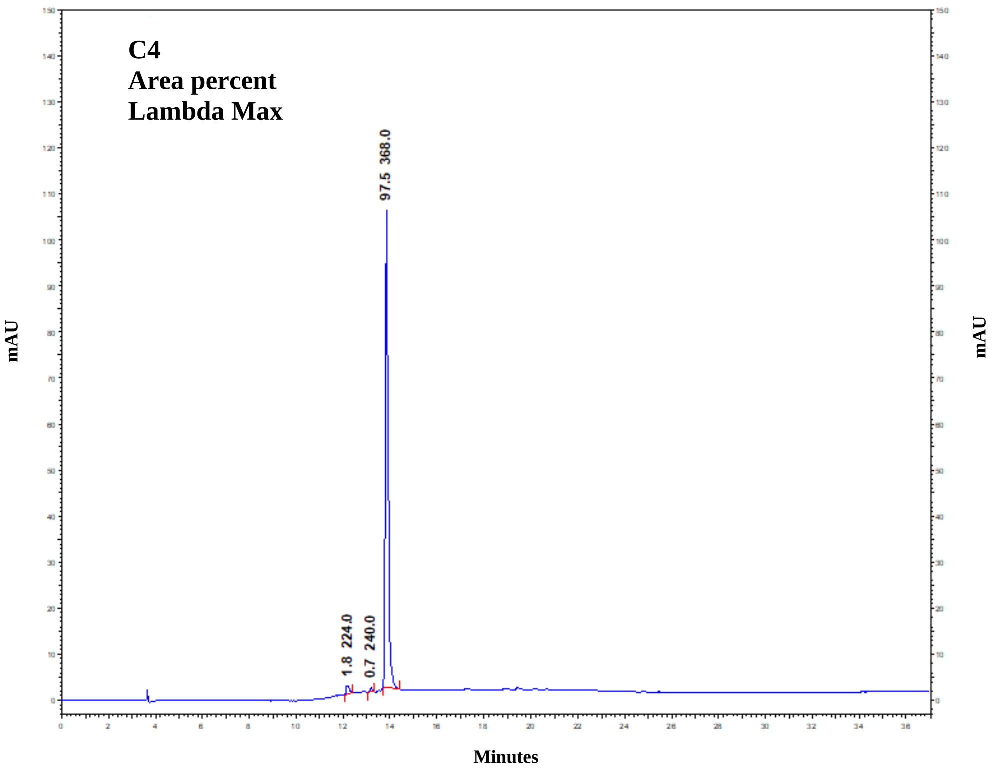

Figure 48. HPLC-DAD: 97.5% purity, with a λmax of 368.0 nm as a result of scanning from 190 to 600 nm of 1-Methyl-*N*'-[(5-nitrofuran-2-yl)methylene]-1*H*-benzimidazole-5-carbohydrazide (**C4**).
